# A multilevel social network approach to studying multiple disease-prevention behaviors

**DOI:** 10.1038/s41598-025-85240-7

**Published:** 2025-01-11

**Authors:** András Vörös, Elisa Bellotti, Carinthia Balabet Nengnong, Mattimi Passah, Quinnie Doreen Nongrum, Charishma Khongwir, Anna Maria van Eijk, Anne Kessler, Rajiv Sarkar, Jane M. Carlton, Sandra Albert

**Affiliations:** 1 School of Social Policy and Society, University of Birmingham, Birmingham, UK; 2 Department of Sociology, University of Manchester, Manchester, UK; 3 Indian Institute of Public Health Shillong, Shillong, Meghalaya India; 4 Martin Luther Christian University, NLHMB, Shillong, Meghalaya India; 5 Center for Genomics and Systems Biology, Department of Biology, New York University, New York, USA; 6 Department of Molecular Microbiology and Immunology, Johns Hopkins Malaria Research Institute, Johns Hopkins Bloomberg School of Public Health, Baltimore, MD 21205 USA

**Keywords:** Diseases, Human behaviour, Public health

## Abstract

The effective prevention of many infectious and non-infectious diseases relies on people concurrently adopting multiple prevention behaviors. Individual characteristics, opinion leaders, and social networks have been found to explain why people take up specific prevention behaviors. However, it remains challenging to understand how these factors shape multiple interdependent behaviors. We propose a multilevel social network framework that allows us to study the effects of individual and social factors on multiple disease prevention behaviors simultaneously. We apply this approach to examine the factors explaining eight malaria prevention behaviors, using unique interview data collected from 1529 individuals in 10 hard-to-reach, malaria-endemic villages in Meghalaya, India in 2020–2022. Statistical network modelling reveals exposure to similar behaviors in one’s social network as the most important factor explaining prevention behaviors. Further, we find that households indirectly shape behaviors as key contexts for social ties. Together, these two factors are crucial for explaining the observed patterns of behaviors and social networks in the data, outweighing individual characteristics, opinion leaders, and social network size. The results highlight that social network processes may facilitate or hamper disease prevention efforts that rely on a combination of behaviors. Our approach is well suited to study these processes in the context of various diseases.

## Introduction

Effective disease prevention typically requires people to take up several prevention behaviors at once. This applies to many infectious and non-communicable diseases, even though specific behaviors and approaches to prevention may differ by disease. To curb the spread of airborne infectious diseases, such as influenza or COVID-19, health agencies may advise a series of preventive practices, including face covering, handwashing, social distancing, and vaccination^1,2^. The prevention of chronic diseases, such as different types of cancer^3^ or cardiovascular disease^4^, rests on the combination of healthy diet, exercise, and the avoidance of smoking and alcohol consumption^5^. Vector-borne diseases, such as malaria, may be prevented by reducing exposure to disease vectors (e.g., mosquitoes) through the simultaneous use of tools such as bed nets, repellent sprays, and vaporizers^6^. For public health interventions to be successful in any of these disease domains, it is important that people concurrently adopt as many of the relevant prevention behaviors as possible^7,8^. Therefore, it is crucial to understand why people adopt or reject different behaviors.

### What explains prevention behaviors? Carry-over, individual factors, and social networks

Research on multiple health behavior change (MHBC)^9^ in the context of chronic disease prevention has shown that the success of behavioral interventions is not self-evident. The different behaviors of an individual appear to be interdependent and connected through various positive and negative “carry-over” mechanisms^10^. Smoking, for example, frequently co-occurs with low physical activity or high alcohol consumption^11^. In turn, smoking cessation is associated with an increase in physical activity^12^ and decrease in alcohol consumption^13^. MHBC research posits that adopting a healthy behavior (e.g., stopping smoking) can increase the likelihood of taking up others (e.g., exercising more and drinking less)^14^. This may happen through the transfer^15^, or spill-over^16^, of the individuals’ knowledge, skills, or beliefs regarding the first, “gateway”, behavior^5^. Applied to infectious diseases, such as malaria, behavior carry-over would explain why people who use bed nets to avoid mosquito bites are more likely to adopt repellents too^17^.

However, evidence also suggests compensatory mechanisms between prevention behaviors. For example, long-term smokers may be more active physically if they believe that activity partly compensates for the harmful effects of smoking^5^. Interestingly, similar behavioral patterns have been identified recently in infectious disease prevention. In the case of COVID-19, both transfer and compensatory mechanisms were inferred from correlations between adherence to protective practices and vaccination^1,18^. Compensatory mechanisms may also explain earlier findings that awareness about novel effective HIV therapies were associated with higher levels of risky sexual behaviors^19^. In malaria prevention, people might avoid using repellent creams because they view them as superfluous beyond wearing protective clothing outdoors^20^. Lastly, there is emerging evidence for carry-over mechanisms connecting behaviors across the domains of chronic and infectious disease prevention, such as healthy lifestyle and adherence to COVID-19 protective practices^21^.

Due to the complex links between individual behaviors, it remains a challenge to theoretically and empirically explain why people adopt, or resist, multiple behaviors to prevent a disease. Theories developed to explain single health behaviors have inspired a plethora of research which identified various intra-individual (psychological, demographic) and inter-individual (social) factors^22,23^. For example, studies on malaria prevention have evaluated how individual characteristics such as gender, education, or socio-economic background relate to various preventive and treatment behaviors^17,24^. However, these approaches do not account for carry-over mechanisms and thus may produce misleading findings in cases when people adopt multiple behaviors concurrently. In turn, theories developed specifically for multiple behaviors tend to focus on intra-individual, cognitive and attitudinal, processes that explain behavior change^10,25^, while ignoring key inter-individual ones.

Social networks are increasingly recognized as important in explaining the adoption of single disease prevention behaviors. The size (number of contacts) and composition of one’s network have been shown to matter for prevention behaviors related to HIV^26–28^ and the avoidance of alcohol and smoking^29^. Social influence, adopting a behavior due to exposure to it in one’s network, also plays a role in the uptake of vaccination (e.g., HPV, influenza)^30^, HIV testing^31^, and health-risk behaviors such as smoking, alcohol consumption, and substance use^29,32,33^. Further, contact with health experts and other opinion leaders^34^ appears to shape some prevention behaviors like smoking cessation and abstinence from alcohol^9^, but not others such as vaccination^30^. These findings and theories highlight the need for an approach that allows us to study how social networks and carry-over mechanisms jointly shape disease prevention behaviors, accounting for individual differences.

### Multilevel social networks as a single framework to study multiple prevention behaviors

In this paper, we propose a novel theoretical and analytical framework that combines MHBC research with social network analysis^35–37^ to examine how social networks explain multiple disease prevention behaviors. Our approach is theoretically innovative because it synthesizes insights from MHBC and social influence theories^38–40^, shifting the focus of multiple prevention behavior research from correlation networks of behaviors^41^ to interpersonal social networks. Our framework is also analytically novel because it applies state-of-the-art statistical models for social networks^42,43^ that allow to assess the importance of various factors (e.g., behavior carry-over^9^, individual characteristics^44^, opinion leaders^34^, network size and composition^26–29^, social influence^38–40^) in the adoption of multiple prevention behaviors in real-life settings. This addresses theoretical calls to consider a broad range of factors and utilize empirical data in disease prevention models for infectious and non-communicable diseases^41,45,46^. We demonstrate the potential of our approach in an empirical study of the role of discussion networks about health-related matters in malaria prevention behaviors in ten rural villages in India.

We study the factors explaining multiple disease prevention behaviors in a multilevel social network framework^47^. Multilevel networks have been successfully applied to study economic markets, organizations, international relations, and scientific collaboration^47^, but their potential in the context of disease prevention is yet to be exploited. In a single-level social network approach, the patterns of individuals’ relationships to each other are used to explain a single behavior. For example, the number of friends and family members with positive vaccination attitudes may explain someone’s own attitudes, and decision, to get a specific vaccine^30^. Such exposure to a behavior in one’s network^38^ is thought to represent channels for social influence^48^. The term “influence” in this line of research collectively refers to a range of interpersonal processes through which the behavior of one’s social ties change one’s own behavior^49^. Importantly, this includes influence based on information sharing and normative pressures^50^.

Sociological studies have shown that social influence can lead to behavioral cascades in networks through a process labelled as social contagion^39,40^. When behavior change is triggered by information that diffuses in a network (e.g., about a new vaccine), the model of simple contagion suggests that contact with a single carrier of information is sufficient to adopt a new behavior^39^. However, most behaviors are also subject to normative pressures from others. In this case, complex contagion offers a more realistic model: the adoption of a behavior will require reinforcement by multiple network connections^39^. This happens most efficiently in closely-knit networks, which tend to exist when people share foci of activities^51^ or interact with similar others^52–54^. In turn, network structure is known to be shaped by several factors, such as tie selection based on individual charactersitics^44^.

In contrast to single-level networks, multilevel networks represent more complex social systems in which individuals may be connected to each other through social ties (e.g., regular health-related discussions) and may also be linked to multiple social objects such as events, attitudes, behaviors (e.g., by adopting prevention behaviors). This approach is suitable to explain several prevention behaviors of an individual with patterns of health-related discussion ties and exposure to these behaviors in their social network. Drawing on sociological theories of social influence, we assume that exposure to behaviors in one’s network represents channels for normative pressures. Thus, the effect of network exposure can be understood as a complex contagion process^39,40^, where both the number of network connections to others with specific behaviors and the structure of networks affect the adoption of behaviors. In turn, we primarily view health experts and opinion leaders as sources of information and expertise. We expect their effects on behavior to be akin to simple contagion^39^, where having a single discussion tie to an expert may have an impact^34^.

**Fig. 1:**
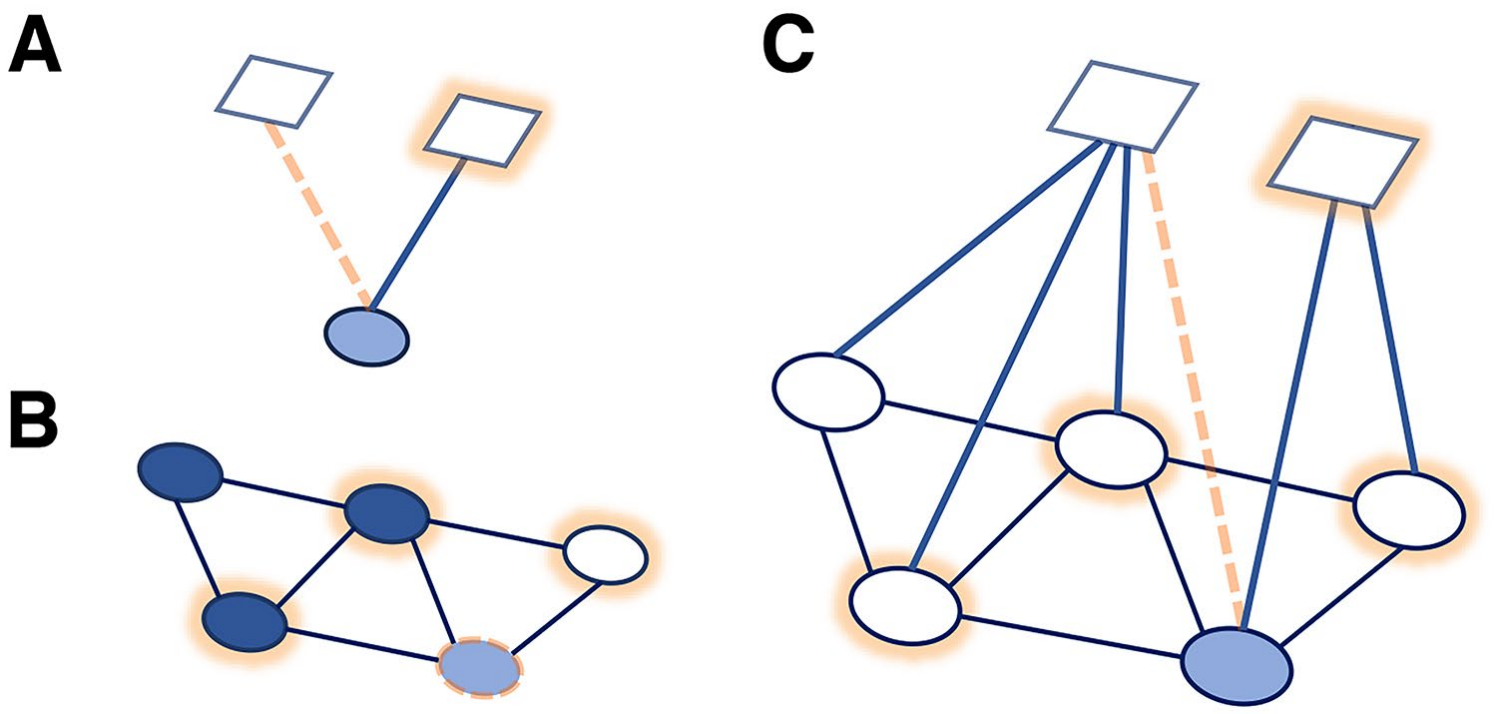
Behavior carry-over and social influence in social networks. In the graphs, circles denote individuals, links between them social ties; squares represent prevention behaviors, links pointing to them the adoption of behaviors by a given individual; dashed lines indicate an individual (the light blue circle) considering to adopt a prevention behavior; their choice may depend on the behavior of the others and their own current behaviors, as shown by highlighted circles and squares. (**A**) *Behavior carry-over mechanisms* suggest that the likelihood of an individual adopting a prevention behavior depends on their *other prevention behaviors*, regardless of the behaviors of other individuals (this would not necessitate a network approach but can be depicted as a two-mode network); (**B**) *Social influence in single-level networks* assumes that a single prevention behavior spreads through a network, with dark blue nodes already adopting it: the likelihood of an individual adopting a behavior depends on their *network exposure* to it (this approach does not consider other behaviors and can be represented as a one-mode network); (**C**) A *multilevel social network* framework is suitable to represent and model both *carry-over* and *social influence* in the context of multiple behaviors (by combining two-mode and one-mode network representations).

Figure 1 shows three network graphs to contrast the discussed theoretical approaches to explaining prevention behaviors: carry-over mechanisms (A), single-level social network influence (B), and multilevel social network influence (C, our approach). In Fig. 1A, an individual following one prevention behavior may be more (or less) likely to adopt a second behavior due to positive (or negative) carry-over mechanisms^5^. In Fig. 1B, an individual connected to others who adopt a single behavior may be more likely to adopt the same behavior due to social influence, also known as network exposure^38–40^. Lastly, Fig. 1C synthesizes the first two approaches and shows that an individual connected to others who adopt multiple behaviors may be more (or less) likely to also adopt certain behaviors due to both carry-over mechanisms and social influence. Who individuals are, in terms of their individual characteristics, their roles as health experts or other opinion leaders, and the structure of their social networks may impact their behaviors and their influence on others.

### The case of malaria prevention behaviors in rural villages of Meghalaya, India

In the rest of the paper, we test our novel theoretical and analytical approach in the context of malaria, a disease that has been on the decline in the past two decades owing to global public health efforts^55^. Large-scale household interventions promoting certain prevention behaviors, such as Indoor Residual Spraying (IRS) and the use of Long-Lasting Insecticidal Nets (LLINs) for beds, played a pivotal role in controlling and eliminating malaria in some countries^56,57^. However, the effectiveness of public health initiatives has varied across the malarious world^56–58^. In particular, they have resulted in little change in poor, migrant, and hard-to-reach populations^55^. These residual epicenters continue to present a challenge to ongoing eradication efforts. Prevention in such contexts requires targeted approaches that facilitate the use of several techniques to reduce mosquito bites^55,59,60^. Targeted interventions are undermined by a lack of understanding of why people adopt certain prevention behaviors but resist others^17,20,61^.

We assess the importance of various individual characteristics, health experts, and social network factors (size, composition, exposure) in explaining the use of eight malaria mosquito-bite preventive techniques in hard-to-reach tribal communities in Meghalaya state of Northeast India. We collected unique interview data about prevention behaviors, health-related discussion networks, individual characteristics, and household membership from every reachable adult in ten villages (n = 1529 individuals) between January 2020 and August 2022. For details about sampling see the Methods section and section 1.1 of the Supplementary Information. Based on prior research^6,62–65^ and our research team’s previous epidemiological studies in different regions of India^17^, including Meghalaya^66^, we identified eight preventive techniques that were well-known among villagers: LLINs, covering clothes, boots, gloves, insecticide cream, coils, vaporizers, and burning materials (e.g., egg boxes). Further, we spoke to two types of health experts in the villages: government-employed Accredited Social Health Activists (ASHAs), who represent the approach of modern medicine, and Traditional Healers^67^, who advocate largely undocumented tribal prevention and treatment methods. Both experts can be considered opinion leaders in the context of malaria prevention due to their expertise, provision of healthcare, and social network position in the villages (see section 1.3 of the Supplementary Information for further details).

**Fig. 2:**
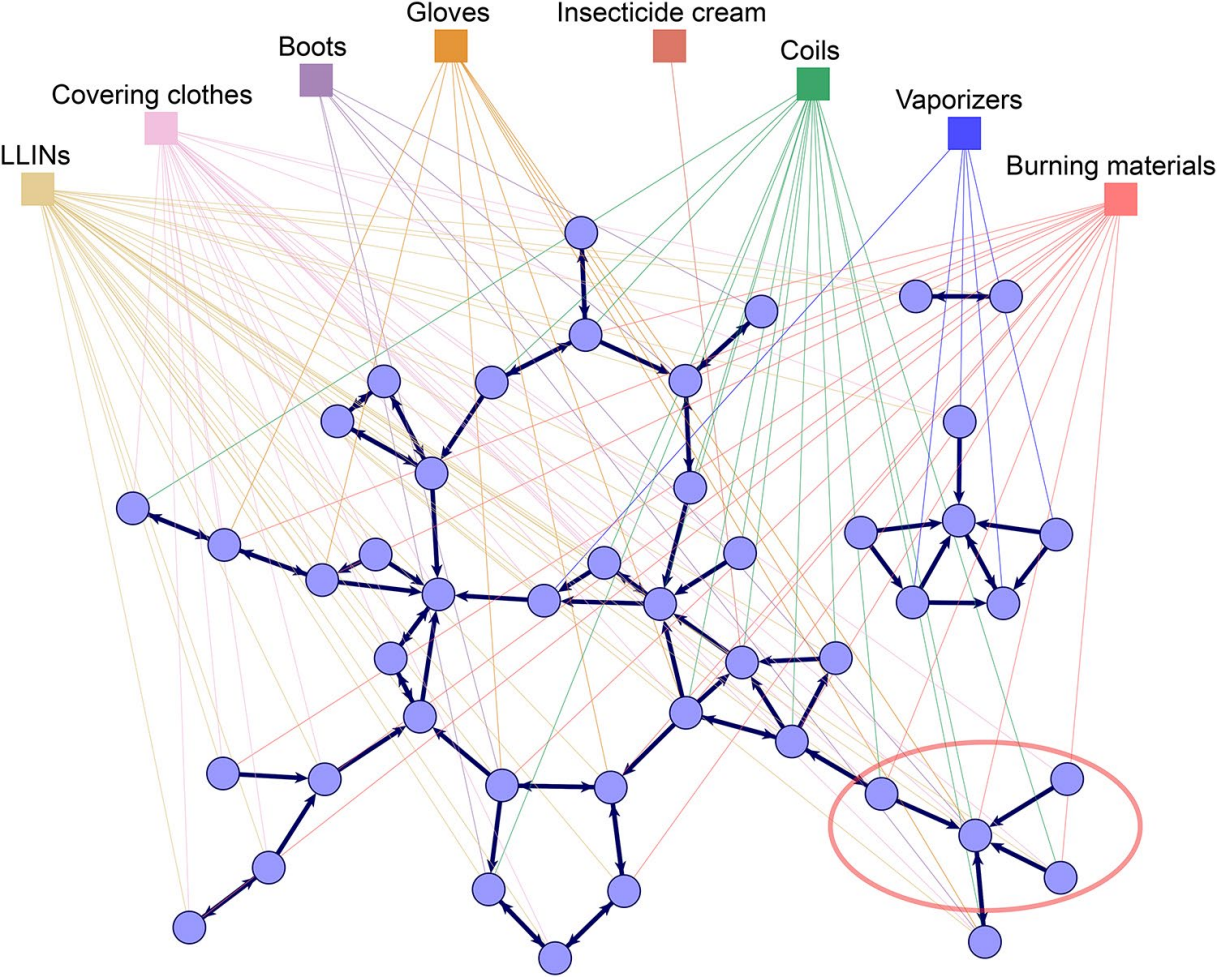
The multilevel social network of health-related discussions and malaria prevention behaviors in one of the studied villages. Circles represent the 46 interviewed villagers (excluding village health experts—see the Methods section for details), squares represent the eight preventive behaviors considered (LLINS—long-lasting insecticidal nets for beds); links between villagers represent health-related discussion ties as reported by the sender of the tie; links between villagers and prevention behaviors represent the reported adoption of the given behavior. For example, the villagers in the bottom right corner of the graph, circled in red, talk to each other and tend to use coils and burn materials. The graph was generated in the Visone software^68^; for comparable visualizations of each of the ten village networks, see section 3 of the Supplementary Information.

The multilevel social network of health-related discussions and adoption of prevention behaviors observed in one of the studied villages is visualized in Fig. 2. The graph represents health-related discussion ties between respondents (first level) as thick dark blue lines with arrows pointing from respondents to their reported discussion partners. It also shows connections from respondents to the prevention behaviors they adopt (second level) as thin lines colored by behavior. Figure 2 demonstrates the differential prevalence of prevention behaviors in this village: for instance, LLINs and covering clothes are used by many, while vaporizers and insecticide cream are used by only a few people. We can further see examples in the figure where individuals are connected by discussion ties and adopt similar behaviors, such the villagers in the bottom right corner of the graph, circled in red, who talk to each other, use coils, and burn materials. Is this pattern explained by carry-over mechanisms, social network influence, or other social or individual factors? To answer this question, we define a similar network graph for all ten studied villages and analyze these statistically.

### Studying multilevel networks in villages using stochastic actor-oriented models

To examine how health-related discussion networks explain malaria prevention behaviors in the ten studied villages, we need a statistical model that can simultaneously consider the impact of carry-over mechanisms, individual characteristics, health experts, network size and composition, and network exposure on multiple behaviors. Very few methods proposed to date meet all these requirements. Two prominent analytical approaches are the stochastic actor-oriented model (SAOM)^43^ and the exponential random graph model (ERGM)^69^. Here we apply the SAOM^42,70^, which combines statistical estimation and agent-based simulations to assess which individual and social factors explain the multilevel network of prevention behaviors and discussion ties. Even though SAOMs are traditionally associated with longitudinal data and ERGMs with cross-sectional data, recent extensions of each model have allowed to overcome this distinction^71^. In fact, it has been shown that when applied to cross-sectional data, the SAOM and the ERGM share some of their key assumptions and limitations^71^. We chose the SAOM in our study as the actor-oriented nature of the model provides a more straightforward interpretation of results than the tie-oriented ERGM, with reference to individuals deciding to have (or not to have) health-related discussion ties with others, as well as deciding to adopt (or not to adopt) prevention behaviors^72^. Apart from the difference in actor- and tie-oriented interpretations^71^, the two models are equally appropriate for the present application.

Analytically, we use a cross-sectional (or “stationary”) version of the SAOM that was developed to explain patterns in networks that were observed at one time point. Stationary SAOMs have been scarcely applied in the literature to date, and to our knowledge never in the context of disease prevention^73,74^. The model assumes that our village networks are in a short-term dynamic equilibrium: social ties and prevention behaviors may change, but these changes do not alter the overall structure of the networks^73,74^. We note that this does not mean that the real-world networks are expected to be wholly static, only that some of their structural characteristics remain stable over a short period of time (an assumption also necessary for interpreting ERGMs^75^). We cannot verify this assumption empirically as we did not gather longitudinal data, but it appears reasonable based on our field experience in the villages and how people spoke to us about health-related discussions and behaviors (for further details, see the Methods section and section 4.6 of the Supplementary Information). Due to the mechanistic nature of the model, we can identify different competing explanations for the adoption of prevention behaviors and thus make inference to the generative processes shaping behavior adoption^76^. The model is empirically calibrated and its fit to the underlying data can be assessed to ensure that the identified explanatory factors, altogether, provide a reasonable explanation for the observed network^77^.

In the following, we summarize our analytic strategy. We provide further details about our study design and analysis approach in the Methods section. In our multilevel network framework the SAOM seeks to explain the probability that villagers adopt prevention behaviors and have discussion ties to others by the network configurations these behaviors and ties are embedded in. The concurrent modelling of behavior adoption and discussion ties is a key feature of our model, as this allows us to account for the factors shaping the discussion network when we consider its impact on prevention behaviors^43,44^. This is technically achieved by specifying two sets of explanatory variables, also referred to as “effects”^42^: one set explaining prevention behaviors, the other explaining health-related discussion ties. For example, model effects may express the prevalence of a prevention behavior in the village, as the number of villagers adopting it, or the popularity of a villager in the village discussion network, as the number of other villagers who report talking to them. Model parameters are estimated for each effect to represent their impact on the probability of a villager adopting a given behavior or having a specific discussion tie to someone else. As noted above, in the cross-sectional model, parameters are optimized so that they keep the network structure stable in the short term.

To examine the role of various individual and social factors in prevention behaviors, we need to specify several explanatory effects in the model representing these factors. Behavior carry-over (cf. Fig. 1A) can be expressed as the effect of the number of behaviors pursued by an individual apart from the one the adoption of which is to be explained. This should be interpreted accounting for other effects related to the structure of behaviors, such as the prevalence of the given behavior in the village (the number of individuals adopting it) or trends for individuals to adopt similar sets of behaviors as others (the number of times an individual adopts the same behaviors as others even if they do not talk to them). Individual factors and ties to health experts (the ASHA and Traditional Healer) can be expressed in the model as the effects of a series of covariates defined for individuals. Social network size can be considered as the effect of the number of discussion ties one has on adopting prevention behaviors. Social influence (cf. Fig. 1B,C) can be expressed as network exposure to a behavior: the effect of the number of villagers one talks to who report pursuing a given behavior on the focal individual’s adoption of the same behavior. We define a similar effect that captures influence between those who live in the same household (regardless of having discussion ties to one another), which we label as household exposure.

**Fig. 3:**
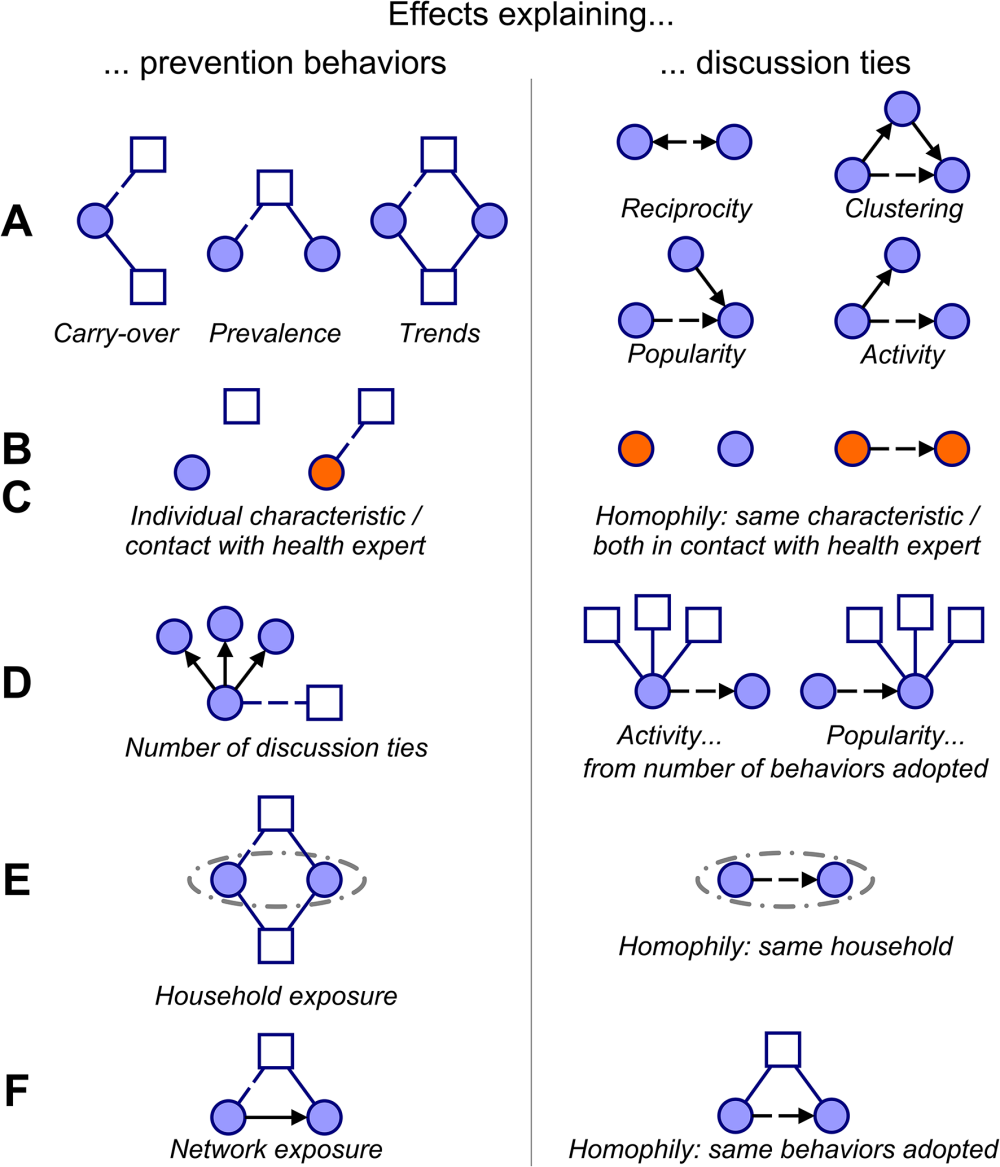
Selection of key SAOM effects representing factors that explain prevention behaviors and discussion ties. Circles represent villagers, squares prevention behaviors. Solid lines indicate the adoption of prevention behaviors (blue) and discussion ties to villagers (black); dashed lines mark the adoption of a behavior or a discussion tie that is explained by the given model effect; arrows represent the direction of discussion ties (the sender reported having health-related discussions with the receiver). The effects can be grouped into six categories based on the explanatory factors they pertain to: (**A**) Network structural factors, (**B**) Individual factors; (**C**) Ties to health experts; (**D**) Network size at the level of discussion ties (to explain behavior adoption) or at the level of behavior adoption (to explain discussion ties); (**E**) Household factors; (**F**) Network exposure. The models include additional effects as some presented here can be defined in multiple ways due to tie directionality or the different individual characteristics and health experts considered. The full list of effects considered in the model is further discussed in the Methods section and presented in section 4 of the Supplementary Information.

Lastly, the network composition of individuals may be a result of various network selection mechanisms^54,78^ that shape discussion ties. These include reciprocity (forming mutual ties), clustering (becoming part of densely connected groups), popularity (being named as health discussion partner by many respondents), activity (naming many others as discussion partners), and homophily (having ties to others who have similar individual characteristics, e.g. gender, who belong to the same household, who have similar ties, e.g. talking to health experts, or who pursue similar prevention behaviors)^32,33^. These represent important alternative explanations for the above effects on prevention behaviors that need to be explicitly modelled. For example, a tendency to talk to villagers who adopt the same prevention behaviors as oneself leads to the same network patterns as social influence: those connected by social ties adopt the same behaviors. In our context, popularity and activity in the discussion network may be affected by the number of prevention behaviors adopted, by adopting similar prevention behaviors as others, by having similar individual characteristics (e.g., gender), and by living in the same household. With the inclusion of a wide range of explanatory factors in our model, we can simultaneously assess their roles in shaping prevention behaviors. Figure 3 presents an overview of the effects pertaining to key explanatory factors with intuitive visualizations.

We apply our modelling approach to the ten village networks in multiple steps. First, we fit a SAOM to each village’s data using the same model specification that includes the above key model effects. Second, we combine the results in a parameter-wise meta-analysis and report the overall estimates and their estimated uncertainty. These results give an insight into which factors shape prevention behaviors consistently across the villages. Finally, we compare the goodness of fit of different nested model specifications across the ten datasets, adding distinct sets of factors (individual characteristics, ties to health experts, network size, household exposure, network exposure) sequentially. This allows us to assess the importance of the different factors in explaining the observed patterns of behaviors and discussion ties.

## Results

On average across all ten villages, participants report adopting three of the eight prevention behaviors. As Table 1 shows, some behaviors are adopted more commonly than others: using LLINs were mentioned by 95% of respondents, using gloves by only 4%, with the rest varying in-between. Health-related discussion ties are descriptively associated with prevention behaviors: participants report adopting a behavior in 38% of the cases if someone they talk to also reports adopting it, and in 31% if nobody they talk to does.

**Table 1:** Summary statistics about the prevalence of the eight prevention behaviors in the studied villages.

	LLINS (%)	Covering clothes (%)	Boots (%)	Gloves (%)	Insecticide cream (%)	Coils (%)	Vaporizers (%)	Burning materials (%)
Average	95	61	22	4	13	66	9	22
Standard deviation	7	14	13	8	8	19	7	13
Minimum	80	36	3	0	2	33	0	3
Maximum	100	80	49	20	25	86	22	49

Percentages refer to proportion of respondents per village who reported adopting a given behavior.

**Fig. 4:**
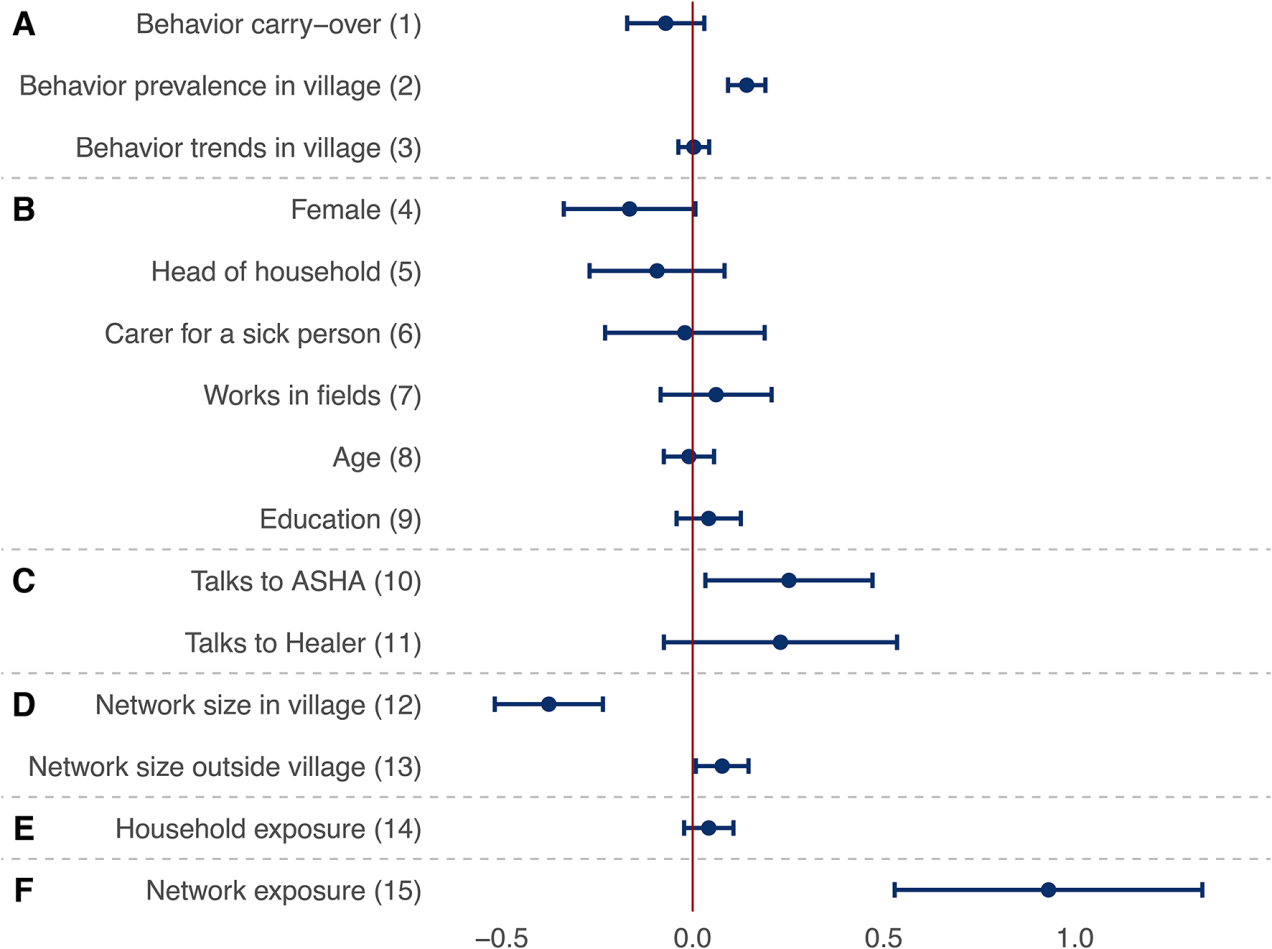
Factors explaining the adoption of malaria prevention behaviors. Each row presents a parameter (solid circle) and confidence interval (horizontal bar) from a meta-analysis of identically-specified stochastic actor-oriented models fitted in each village; effect groups A–F are identical to those shown in Fig. 3; estimates can be interpreted as log-odds ratios of the use of a given behavior; these parameters were simultaneously estimated with those presented in Fig. 5; for further control variables in the models as well as heterogeneity statistics for the meta-analyses, see section 4 of the Supplementary Information.

**Fig. 5:**
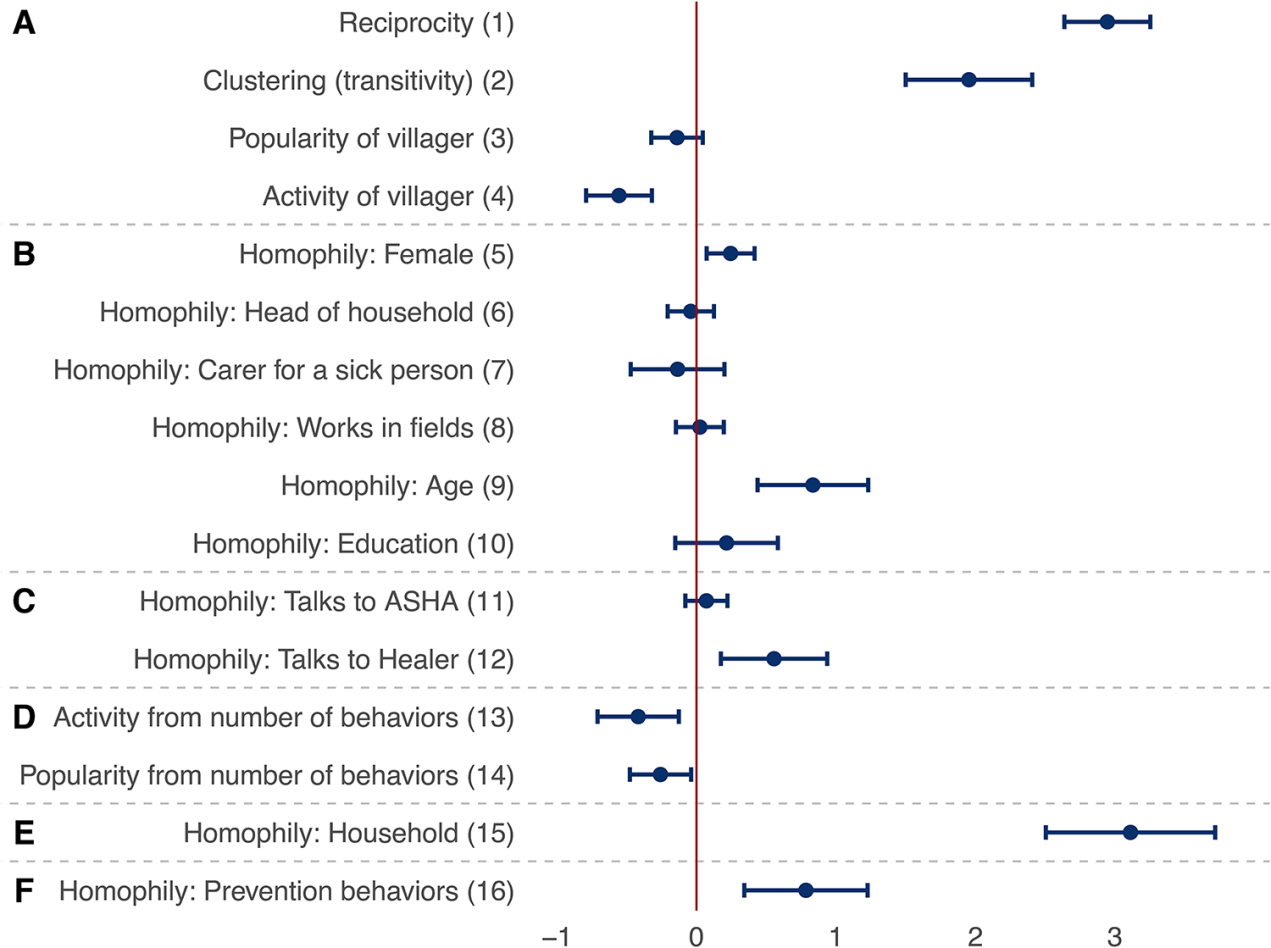
Factors explaining health-related discussion ties. Each row presents a parameter (solid circle) and confidence interval (horizontal bar) from a meta-analysis of identically-specified stochastic actor-oriented models fitted in each village; effect groups A–F are identical to those shown in Fig. 3; estimates are interpreted as log-odds ratios of a discussion tie being sent to a given other villager; these parameters were simultaneously estimated with those presented in Fig. 4; for further explanatory variables in the models as well as heterogeneity statistics for the meta-analyses, see section 4 of the Supplementary Information.

Figures 4 and 5 present the key results from the meta-analysis of the multilevel SAOMs fitted in each of the ten villages, reporting the factors that may explain the patterns of adoption of prevention behaviors and discussion ties. Estimates are interpreted as log-odds ratios, akin to logistic regression, and refer to the contribution of a given effect statistic to the probability of a villager adopting a prevention behavior (Fig. 4) or reporting health-related discussions with another villager (Fig. 5). The presented results are weighted aggregates from ten village-level models (see the Methods section for details).

### Which factors explain malaria prevention behaviors?

First, we discuss the factors explaining the adoption of the eight prevention behaviors based on Fig. 4. The 15 factors can be grouped as effects of the structure of behaviors (parameters 1–3 in the figure), individual characteristics (4–9), ties to health experts (10–11), discussion network size (12–13), household exposure (14), and discussion network exposure (15). The full models include further control variables, which are listed in section 4 of the Supplementary Information. Behavior structural effects (parameters 1–3 in the figure) show no evidence for behavior carry-over (parameter 1). This is found while accounting for the fact that some behaviors (like using LLINs) are more prevalent than others (parameter 2) and that there do not appear to be trends among villagers of following the same set of behaviors (parameter 3).

Individual factors (parameters 4–9) do not systematically relate to behavior across the villages. There is some evidence for the relevance of health experts (parameters 10–11): those talking to the village ASHA, who is trained in modern medicine, are more likely to use preventive techniques in general (further variables not included in this table show no additional effect for specific techniques used by the ASHA—see section 4 of the Supplementary Information for details). Network size (parameters 12–13) appears to matter in two ways: having more discussion partners from one’s own village reduces the probability of engaging in malaria prevention behaviors (parameter 12), while more contacts outside the village increases it (parameter 13).

Exposure to prevention behaviors in households does not have a significant effect on one’s own behavior (parameter 14). Finally, and most importantly, we find clear evidence for social influence (parameter 15) in that being exposed to a prevention behavior in one’s discussion network increases the chances of adopting the same behavior (odds ratio: 2.5 for each additional connection that adopts the behavior). These findings show that social networks, and especially network exposure, have more consistent effects on prevention behaviors than behavior carry-over, individual factors, or health experts.

### The structure and composition of health-related discussion networks

Figure 5 presents factors explaining the structure of the discussion network whose effects were simultaneously estimated with the results related to behavioral adoption (Fig. 4). As noted earlier, this is a key feature in our analysis, as network selection mechanisms, such as reciprocity, clustering, or homophily, may confound the effects of the factors examined for behavioral adoption. The 16 estimates presented here explain health-related discussion ties, but they can be grouped similarly as earlier as effects of discussion network structure (parameters 1–4), individual characteristics (5–10), ties to health experts (11–12), engagement in multiple prevention behaviors (“behavior network size”) (13–14), being in the same household (15), and similarity in adoption of prevention behaviors (16). The full models include further control variables explaining discussion ties, which are listed in section 4 of the Supplementary Information.

Figure 5 shows that reciprocity and clustering both shape network structure (parameters 1–2) as well as a tendency for villagers to report few connections (parameter 4). This suggests that health-related discussions are structured similarly to other relational networks, such as friendships, in which individuals have relatively few but closely-knit ties to others^42^. Regarding homophily based on individual characteristics (parameters 5–10), villagers tend to talk to others who are of the same gender (parameter 5) and similar age (parameter 9) as themselves. Similarity in talking to health experts (parameters 11–12) further affects network structure, with those talking to the Traditional Healer of their village being more likely to talk to each other (parameter 12). Engagement in prevention behaviors shapes network structure as well (parameters 13–14) in that those who adopt more behaviors tend to be connected to fewer villagers. These effects contribute to villagers’ discussion network size further to the structural effects discussed above. Importantly, there is a strong tendency for health-related discussions to be reported within households (parameter 15, odds ratio: 22.4). Lastly, people are more likely to talk to those who pursue the same behaviors as they do (parameter 16, odds ratio: 2.2 for each additional behavior). Overall, these results suggest that a number of social network mechanisms may shape villagers’ discussion networks. It is crucial to take these into account when explaining prevention behaviors.

### Network exposure and household discussions explain patterns of prevention behaviors and social ties

**Fig. 6:**
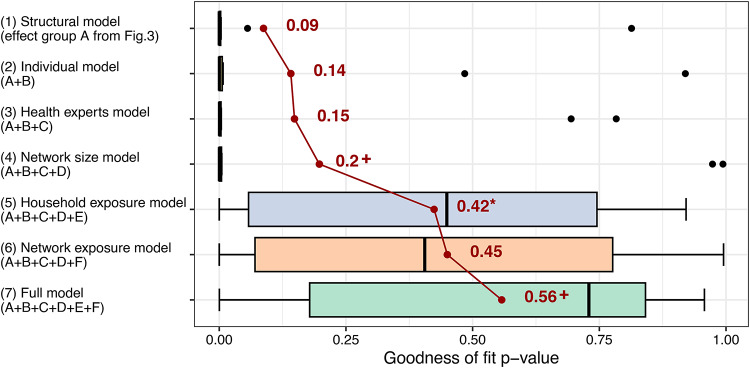
Importance of different factors in explaining the observed patterns of preventive behaviors and health-related discussions. The bar charts represent the goodness of fit of different stochastic actor-oriented model specifications; each bar shows the distribution of model fit in ten village-level multilevel network models; higher values the goodness of fit *p* value indicate better model fit on the mixed triad census^79^; model fit calculations were based on 5000 simulations from each village-level model; the average level of fit for each model is shown by the connected red points and corresponding numbers; the */+ highlight that the addition of a set of factors significantly improved model fit beyond the previous model at 5%/10% significance; for further details about the assessment of model fit, see the section 4.5 of the Supplementary Information.

Lastly, to assess the importance of different factors in explaining the interrelated patterns of behavior and discussion ties, we compared the contribution of seven sets of parameters to the fit of the multilevel network model to the observed data. Due to the characteristics of the model, model fit can be tested through simulations^77^. Figure 6 shows the distribution of model fit statistics in the ten villages with regard to the mixed triad census of the two modelled networks^79^. A higher model fit *p* value signals a closer fit between the model and the observed networks. We found that a model considering only the effects of network structure (Model 1) for discussions and behaviors (first set of parameters in Figs. 4 and 5) generally achieved a poor fit on the considered statistics (mean = 0.09; one sample t-test with two-sided *p* value: t = 1.08, df = 9, *p* = 0.308). Sequentially adding effects of individual characteristics (Model 2) and health experts (Model 3) did not significantly improve model fit. Further adding network size (Model 4) led to a small improvement which was not significant at 5% (difference = 0.05, t = 1.49, df = 9, *p* = 0.085).

Adding the effects of exposure and discussions in households (Model 5) to the previous model led to a substantial improvement in average model fit of 0.23 (paired sample t-test with one-sided *p* values: t = 2.34, df = 9, *p* = 0.022). The same was true if we instead add network exposure and selection based on similar behavior to Model 4 (Model 6) which led to a somewhat larger improvement of 0.25 (t = 2.60, df = 9, *p* = 0.014) over the network size model. The inclusion of both household and network exposure effects in the Full model (Model 7) leads to improved average model fit, but the improvement is not significant at 5% over the household exposure model (difference = 0.13, t = 1.72, df = 9, *p* = 0.060) or the network exposure model (difference = 0.11, t = 1.48, df = 9, *p* = 0.087).

Overall, these results indicate that network exposure and discussion ties within households are important in explaining the links between multiple prevention behaviors and discussion networks, while carry-over and other network structural factors, individual characteristics, health experts, and network size are not. As described previously, it is network exposure that is positively associated with behavior adoption (Fig. 4). At the same time, households emerge as relevant in explaining the structure of the health-related discussion network (Fig. 5).

## Discussion

In this paper, we presented a novel approach to studying multiple disease-prevention behaviors. Drawing on research on multiple health behavior change (MHBC)^5,9^, social influence^38–40,48–50^, and statistical network modelling^42,73^, we proposed to consider these behaviors embedded in their social context, as part of a multilevel social network^47^. In these networks, people are connected to each other by social ties and to behaviors based on whether they pursue them. Theoretically, multilevel networks are uniquely suited to represent multiple prevention behaviors as outcomes of several mechanisms that were found relevant in prior research, such as behavior carry-over^10^ and social influence^38–40^. Analytically, our approach allowed us to draw on recent advances in statistical network modelling^69,73^ to study disease-prevention behaviors. We used one of the few suitable methods, the stochastic actor-oriented model^42,43^, to examine the impact of a wide range of factors, including individual characteristics, health experts, and social networks, on multiple behaviors. Our approach contributes to the broad area of disease prevention in two ways. First, it further integrates the study of social and individual factors in health behaviors by synthesizing insights from MHBC and social network influence theories^39,40,48^. Second, it provides a viable and practical method for studying a broad range of factors that explain multiple prevention behaviors in a single, empirically-informed modelling framework, which can be used in the context various infectious and non-communicable diseases^41,45^.

We applied our approach to unique empirical data we collected about malaria-prevention behaviors in 10 remote, malaria-endemic villages of Meghalaya state in India. Our analysis identified exposure to behaviors in one’s social network as the most important factor that explains prevention behaviors in this context, while household membership was key in shaping health-related discussions. Crucially, we did not find evidence for behavior carry-over: villagers did not appear to adopt prevention behaviors in bundles, but they would adopt behaviors that were followed by the people they talked to about health-related matters. In turn, these health discussion ties tended to be mutual, clustered, and exist between people of the same household, same gender, similar age, and with similar prevention behaviors. Interestingly, we did not find evidence that the considered individual characteristics or health experts would substantially explain prevention behaviors.

The results suggest that inter-individual processes, such as social influence and network selection, may have had a larger impact on prevention behaviors in these villages than intra-individual processes, such as carry-over or effects of individual characteristics. These findings may inform public health interventions which, in the case of malaria, have focused mainly on the mass-distribution of prevention tools^60,61^ and relied on local health experts^67^. Our study suggests that leveraging the influence of social ties on prevention behavior could complement and increase the effectiveness of centralized prevention efforts. Individual factors and health experts may still play an important role in this as they shape the structure of social networks, which in turn affects the uptake of behaviors.

The distinct findings regarding network exposure and ties to health experts highlight that different types of interpersonal influence (informational vs. normative)^50^ and network contagion (simple vs. complex)^39^ processes may operate concurrently in real-life social settings. The importance of network exposure suggests that prevention behaviors may require normative reinforcement from social ties, which is characteristic of complex contagion. At the same time, the minor effect of single discussion ties to health experts, in our case the ASHA, points to the potential role of influence through information and health expertise in the spread of behaviors. These findings underline the future need for rich empirical data^41,45,46^ and multi-mechanistic models^78^ to better understand how different social influence mechanisms shape disease prevention behaviors.

Our study highlights that we should understand single health behaviors as parts of a wider social-behavioral system. However, this should not come at the neglect of exploring intra-individual mechanisms. While we did not find evidence for effects of behavior carry-over and individual characteristics, our data did not allow us to fully explore these factors. Why someone adopts or resists a prevention behavior, such as vaccination against a specific disease^30^, may depend on a host of related behaviors, beliefs, and attitudes of the individual^2,5^. Further, carry-over mechanisms may operate distinctly for specific subsets of behaviors. For instance, villagers in our sample may avoid using insecticide creams if they are already wearing covering clothes outdoors^20^. Although these processes were beyond the scope of this paper, our approach can be naturally extended to study health beliefs and behaviors across the domains of multiple, infectious and chronic, diseases^21^ and to distinguish processes specific to certain clusters of behaviors^41^. Such extensions could readily be implemented in the statistical method used in our analysis^43^.

We only had modest resources to observe a non-representative sample of villages in Meghalaya, and so our specific findings do not generalize to the state or other regions. However, our research informs future large-scale studies into the social determinants of disease prevention and other health behaviors. Our finding about the role of households in structuring discussion networks suggests that future work should explore the key contexts where people exchange information and influence each other’s health behaviors. In larger or urban settings, various face-to-face and online communities may serve this role besides households. By mapping relevant meeting foci^51^ and combining these with social network data, researchers can more accurately measure and model the dynamics of networks and health behaviors.

Exploring socializing contexts can also facilitate the scale-up of multilevel network data collection in large communities. Focusing on offline and online meeting places may enable the use of various data collection methods, including field observations^80,81^, smart sensor technologies^82–84^, and digital surveys. These may complement traditional face-to-face interviews and provide insight into the content and context of social ties and prevention behaviors. Interviews were appropriate for the relatively small village communities we studied. They helped us establish trust with participants, reach adequate response rates, and elicit meaningful responses. More intrusive methods like the above may lead to lower participation rates^84^ or altered behavior^85^. These considerations suggest that large-scale studies into social networks and prevention behaviors should rely on a combination of established and emerging data collection techniques.

We interpreted our key results in terms of social processes, while we collected and analyzed cross-sectional data. We do not have information about how stable prevention behaviors or health-related discussion ties were over time in the ten villages, although our experience in the field and the social network literature suggest that short-term stability is a reasonable assumption^86^. Despite this limitation, our statistical modelling approach is suitable to identify the social processes at play. The stochastic-actor oriented model assumes that villagers may still change their behavior and network ties even if the observed data is cross-sectional. However, the changes are assumed to keep the overall structure of the multilevel network of each village in a stable state^73^. This way, the model can separate effects of network ties on behaviors and vice versa. By concurrently modelling the interdependencies between network ties and behavior, we can infer the social mechanisms that are likely to maintain the state of the multilevel network system^75^, such as social influence and discussions in households. Ultimately, collecting longitudinal data and studying the actual changes of networks and behavior is necessary to gain a clear understanding of the dynamics of prevention behaviors over time. While this was beyond the scope of our study, it is straightforward to adapt our data collection and analysis approach to a longitudinal design.

For the first time since the start of malaria elimination efforts in India^87^, local elimination may be within reach. However, current control efforts rely solely on mass-administered interventions and include large-scale distribution of LLINS, which was interrupted in India during the COVID-19 pandemic^88^, and IRS, which is met with high levels of refusal across the state^20,61^. Our study suggests that these interventions could be complemented with targeted efforts that rely on social influence in interpersonal relations to increase the uptake of alternative prevention techniques. Such efforts could support downstream elimination goals, including influencing the acceptance of a malaria vaccine. Future studies based on our approach could help to develop network-based strategies to promote multiple prevention behaviors for various infectious and non-communicable diseases. Looking beyond the scope of a single disease, examining a wider set of health behaviors and beliefs could help to understand and exploit links between people’s long-term healthy lifestyles and short-term, targeted prevention behaviors^21^.

## Methods

### Study design and data collection

From January 2020 to August 2022, we collected data from residents of ten villages in Meghalaya state in India: three villages in West Khasi Hills, three in West Jaintia Hills and four in South Garo Hills. The ten villages were selected based on their manageable size (< 500 eligible adults), their known willingness to participate in similar studies^89^, and their accessibility either by car or on foot. As the villages were not randomly sampled, the findings cannot be generalized beyond the studied villages. We administered a structured interview to every reachable adult (18+ years) in each village and gathered responses from 1529 villagers in 764 households. The overall response rate was 68% for individuals and 80% for households (calculated as the proportion of households represented by at least one respondent in the data). Response rates varied by village and ranged from 53 to 88% of residents and from 73 to 97% of households. Individual response rates were below 65% only in two villages. Non-respondents tended to be peripheral in the village communities: many of them were staying away from their village for a longer period due to work or other reasons and they were mentioned much less frequently as health-related discussion partners by others compared to respondents (0.56 vs. 1.85 mentions received on average). This suggests that non-response had a limited effect on the structure of the measured social networks. Further details regarding response rates, characteristics of non-respondents, and the impact of missing data are reported in section 1.2 of the Supplementary Information (Tables S1-S2).

Interviews were bilingual, conducted in the appropriate local language (Khasi, Pnar, or Garo) and the official language of Meghalaya (English). Respondents were asked a series of questions regarding their individual characteristics, household roles, their adoption of malaria prevention behaviors, and health-related discussion ties. The English translation of the specific questions that were asked and frequencies of responses are presented in section 2 of the Supplementary Information (Tables S3-S16). In total, each interview consisted of 26 questions and lasted about 30 minutes. We did not collect information about individual or household malaria infection history, as villagers do not always get tested when experiencing symptoms, so they may not know if they, or their family members, were previously infected^90^.

Questions related to the use of mosquito bite preventive techniques were originally designed by our research team for an earlier epidemiological questionnaire distributed in other areas of India as part of the International Centers for Excellence in Malaria Research CSCMi 1.0 (2010–2017)^17^. The questions were subsequently tested in an epidemiological study in Meghalaya^66^, the site of the current research. These studies did not collect data about social networks. Questions regarding health-related discussions were intentionally asked broadly, without specifying the health topics discussed (such as malaria) or the reference period for discussions (such as occurring in the past week or month). This was to ensure that the village networks constructed from responses contained a sufficiently large number of ties to make them suitable statistical modelling^91^. On average, respondents named 1.85 villagers (sd = 1.15) with whom they discussed health-related matters of any kind (see Table S11 in the Supplementary Information). This rather low number underscores the practical relevance of using a broad definition of social ties in our study.

Due to their role as opinion leaders in health-related matters^67^, we interviewed the Accredited Social Health Activist (ASHA) in all ten villages, and the tribal Traditional Healer in the six villages that had one. The procedure we used to identify the ASHA and the Traditional Healer is detailed in section 1.3 of the Supplementary Information. Once identified, we approached these experts directly in the villages by visiting their homes. We do not use the two types of health experts as respondents in our analyses, but we utilize information about their prevention behaviors and about who talks to them to explain the behavior and social networks of other villagers (see Table S13 in the Supplementary Information). Further details about the data collection, sample size, and questionnaire items are reported in sections 1–2 of the Supplementary Information.

Permission to conduct the study was granted by the Headman of each village, and all respondents signed an individual informed consent form. Identifiers of the villages and individual participants were removed from the dataset and this article to provide full anonymity. Ethical approval for the study was obtained from the Institutional Review Boards (IRBs) of Martin Luther Christian University, Shillong, Meghalaya, India and New York University, New York, NY, USA. For all fieldwork conducted during the COVID-19 pandemic, our team followed the general safety protocols recommended by the Indian government.

### Variables

Our statistical network analyses have two dependent variables: the adoption of prevention behaviors and health-related discussion ties between villagers. The focal dependent variable in our study is the adoption of eight malaria prevention behaviors by participants: Long-Lasting Insecticidal Nets (LLINs) for beds, covering clothes, boots, gloves, insecticide cream, coils, vaporizers, and burning materials. We seek to model patterns of behavior adoption and health-related discussions simultaneously, considering a variety of explanatory factors:

A. Network structure: existing patterns of prevention behaviors or discussion ties.
B. Individual characteristics and roles: gender, being the head of the household, looking after family members when they are sick, working in fields, age, and educational background.
C. Ties to health experts: talking to the ASHA or the Traditional Healer about health-related matters, considering their prevention behavior.
D. Social network size: number of respondents, non-respondents, people outside one’s own village one talks to, number of prevention behaviors adopted.
E. Household exposure: adoption of prevention behaviors in one’s household, talking to someone in one’s household.
F. Social network exposure: adoption of a prevention behavior in one’s network, talking to someone who adopts the same behavior.

Each of these factors may have different effects on behavior adoption and discussion ties. Further, a given factor (e.g., gender) may affect a dependent variable (e.g., discussion ties) in several different ways (e.g. the gender of the tie sender, the gender of the receiver, the gender of both sender and receiver). Due to this, our models include 62 explanatory variables in total. This number is quite typical for statistical network methods that model the complex structure of social networks, including the specific method we use^74^, as explained below. As discussing all the explanatory variables would have complicated the main text without adding proportional new insight, we decided to focus the presentation of the analytical approach to the 16 key model effects presented in Fig. 3 and that of the results to the 31 selected estimates shown in Figs. 4 and 5. These provide a valid representation of our findings in the full models. The complete list of included variables and their definitions are given in section 4.2 of the Supplementary Information (Tables S17 and S18).

### Method I: Meta-analysis of village-level stationary stochastic actor-oriented models

We applied stationary SAOMs^73^ to simultaneously model villagers’ adoption of eight preventive behaviors and their health-related discussion ties. Stationary SAOMs are an extension of the original stochastic actor-oriented model^42,70^ to the analysis of cross-sectional network data. Here, it is assumed that the studied networks are in a short-term dynamic equilibrium: their structure, but not necessarily single network ties, is in a temporarily stable state^73^. Due to their recent implementation, stationary SAOMs have been scarcely applied in the literature to date^73,74^, which warrants a brief comparison with the standard model.

Generally, SAOMs are empirically-calibrated simulation models that aim to identify the relative strength of a set of social mechanisms that *could have* generated an observed network over time. The social mechanisms considered may operate on network ties, individual covariates and pairwise (dyadic) covariates. In a standard longitudinal SAOM, the model is conditional on the first observation of the network^42^. In a stationary SAOM, the initial and final states of the network are identical, and the modelled social processes will, at least stochastically, maintain the existing structure of the network^73^.

To apply stationary SAOMs, we defined a multilevel network^47^ that consists of two interlinked networks in each village: 1) the network of health-related discussions, which is a one-mode social network where villagers A and B are connected by a binary directed tie if A reports talking to B; and 2) the network of preventive behaviors’ adoption, which is a two-mode network where villager A is connected to behavior M if A reports adopting M. It is important to note here the asymmetric role of villagers and prevention behaviors in the model. In a SAOM defined as such only villagers are assumed to have agency (be in control of their ties and behaviors), while behaviors can only be adopted by villagers. An example of the multilevel network for one of the villages is presented in Fig. 2. Similar graph visualizations for all ten villages can be found in section 3 of the Supplementary Information (Figures S2-S6).

We fitted stationary SAOMs using the RSiena package (v1.3.0) in R^91^. We set the rate parameter to 3.0 for both networks in all villages, as this value enabled model convergence and led to good model fit in all cases. This means that villagers were assumed to make, on average, three choices about their discussion ties and three about their prevention behaviors in the agent-based simulations used for parameter estimation in the SAOM. These parameters defined a sufficiently long simulation period, as suggested by the literature^73,74^, given that the average respondent in our sample had fewer than two discussion ties and reported adopting three of the eight prevention behaviors. These descriptive statistics highlight that a rate parameter of 3.0 allows an average villager to change all their discussion ties and behaviors. This would represent a considerable shift in village networks which, based on our field experience in these villages, could take a long time occur in reality. Thus, the chosen rate parameters were deemed large enough for the study context. Nonetheless, we carried out robustness checks with different rate parameters, which led to similar results. Further reasons for the choice of rate parameters and details about robustness checks are presented in section 4.6 and Table S28 in the Supplementary Information.

The full models included all 62 explanatory variables from the six types of factors introduced in the *Variables* section above. As we noted there, this number of variables is not particularly high in similar network models. To put this in context, we should consider that the model effects are defined for each tie and behavior observation. Thus, the model in each village is informed by n(n-1) + 8n (non-independent) data points, where n is the number of individuals in the village and the number eight refers to the number prevention behaviors considered. This highlights that the SAOM estimation is based on an effective sample size which is considerably larger than the number of respondents, allowing the estimation of a larger set of parameters. Full village-level model results are presented in section 4 of the Supplementary Information (Tables S19-S22).

We performed parameter-wise meta-analyses of village-level SAOMs with random-effects models estimated by restricted maximum likelihood using the metafor package (v3.8–1) in R^92^. The estimates referred to in the main text provide information about the mean and standard error of parameters. The full results of the meta-analyses, including heterogeneity statistics, are reported in section 4 of the Supplementary Information (Tables S23-S24). A selection of key results from these tables are presented in Figs. 4 and 5.

While the goodness of fit of statistical network models such as the SAOM cannot be simply assessed by single measures such as the R-squared, it can be tested against various network statistics calculated from the observed network, using simulations^77^. The village-level models that informed the meta-analyses presented in the main text all provided a good fit to the observed data considering nine sets of model fit statistics that are commonly used to evaluate SAOMs^91^. All model fit *p* values were larger than 0 and many were close to 1, suggesting that the observed statistics were situated within the distribution of statistics simulated from the models^91^. Further details about the fit of village-level models are discussed in section 4 of the Supplementary Information (Table S25).

### Method II: Comparison of the explanatory power of nested SAOMs

We compared the explanatory power of the full SAOMs in each village with nested models that contain different subsets of explanatory variables. Based on the six types of explanatory factors described above, we considered the following model specifications (for a complete list of variables, see Tables S17 and S18 in section 4.2 of the Supplementary Information):

1. Structural model: A (network structural factors from the *Variables* section above)
2. Individual model: A + B (network structure, individual factors)
3. Health experts model: A + B + C (network structure, individual characteristics, ties to health experts)
4. Network size model: A + B + C + D (network structure, individual characteristics, ties to health experts, network size)
5. Household exposure model: A + B + C + D + E (network structure, individual characteristics, ties to health experts, network size, household exposure)
6. Network exposure model: A + B + C + D + F (network structure, individual characteristics, ties to health experts, network size, network exposure)
7. Full model: A + B + C + D + E + F (network structure, individual characteristics, ties to health experts, network size, household exposure, network exposure)

For the comparison, we used a set of model fit statistics that represent the interrelated patterns of prevention behaviors and discussion ties. We chose this aspect of model fit as it informs us about how well the models explain the complex structure of the multilevel network analyzed in each village. Specifically, we calculated *p* values of Mahalanobis distances^93^ for a mixed triad census fit test for the different SAOM specifications. The results reflect how well the distribution of simulated networks based on the estimated models represent the mixed triad census^79^ (different tie combinations between two villagers and a behavior) of the observed networks. Conceptually, a good fit is indicated by small differences between simulated and observed counts of all possible network tie configurations that involve two villagers and one prevention behavior. Practically, a *p* value close to 1 signals good fit, one close to 0 poor fit, while a *p* value that is exactly 0 suggests inadequate fit^91^. Calculations in each model are based on 5000 simulations and follow the steps suggested in the network modelling literature^77^. The goodness of fit tests were carried out using the RSiena R package (v1.3.0)^91^. The results from each village are summarized by model specification in Fig. 6. Further details of the goodness of fit analyses can be found in the Supplementary Information (section 4.5, Tables S26-S27).

## Supplementary Information

Supplementary Information.

## Data Availability

The dataset and the R code that can be used to reproduce the analyses reported can be freely accessed on GitFront.io (link: https://gitfront.io/r/user-9304591/2vEGnBLiLyit/Malaria-network-exposure/).

## Code availability

The R code that can be used to reproduce the analyses reported in the paper can be freely accessed on GitFront.io (link: https://gitfront.io/r/user-9304591/2vEGnBLiLyit/Malaria-network-exposure/).
