# Supplementary material for: A multilevel social network approach to studying multiple disease-prevention behaviors
Source: Sci Rep. 2025 Jan 11;15:1718. doi: 10.1038/s41598-025-85240-7 (PMC11724947; doi:10.1038/s41598-025-85240-7)
Supplement: Supplementary file 1 — Supplementary Information. [file 41598_2025_85240_MOESM1_ESM.pdf]

Supplementary Information for

A multilevel social network approach to studying multiple disease-prevention behaviors

András Vörös, Elisa Bellotti, Carinthia Balabet Nengnong, Mattimi Passah, Quinnie Doreen Nongrum, Charishma Khongwir, Anna Maria van Eijk, Anne Kessler, Rajiv Sarkar, Jane M. Carlton, Sandra Albert

Correspondence to: András Vörös, a.voros@bham.ac.uk; Elisa Bellotti, elisa.bellotti@manchester.ac.uk

Summary

This supplement provides methodological background information for the results reported in the article titled “A multilevel social network approach to studying multiple disease-prevention behaviors”. This article is based on a study we conducted in ten “tribal”, hard-to-reach, malaria endemic villages in three regions of Meghalaya, India in 2020-22. We first provide details about the sampling strategy and empirical data collection (section 1). This is followed by a discussion of the specific questionnaire items and variables used in the reported analyses (section 2). We then present graph visualizations of the ten multilevel social networks of participants and the multiple prevention behaviors they adopt in each village (section 3). These networks are analyzed using a meta-analysis of village-level stochastic actor-oriented models (SAOMs) in the main text. Village networks consist of two types of links which represent health-related discussion ties between respondents and the adoption of different malaria prevention behaviors by respondents. Lastly, we present background information and technical details for the SAOMs we use in the main text to study the social mechanisms linking prevention behaviors and health-related discussion ties (section 4).

Table of Contents

1 Sampling and data collection 2
- 1.1 Sample selection and sampling frame 2
- 1.2 Total sample size and response rates 2
- 1.3 Analytical sample size: use of data collected from village health experts 3
- 1.4 Research ethics 4

2 Questionnaire and variables 4
- 2.1 Adoption of prevention behaviors 4
- 2.2 Individual characteristics 4
- 2.3 Social networks and households 5

3 Visualization of village-level behavior adoption and discussion networks 6

4 Details of the analyses using stochastic actor-oriented models (SAOMs) 7
- 4.1 Overview of modelling approach 7
- 4.2 Model specification and definition of effects 8
- 4.3 Village-level SAOM results with score-type tests 8
- 4.4 Full SAOM meta-analysis results 8
- 4.5 Assessment of the goodness of fit of village-level SAOMs 10
- 4.6 Robustness checks with different SAOM rate parameters 11

5 Figures 12

6 Tables 18

7 References 42

1 Sampling and data collection

This section provides essential details about the sampling and data collection procedure for the study. First, we discuss the selection of the ten studied villages and how the sampling frame of individuals was identified in each village. We then derive the total sample size and examine response rates across villages. We proceed by discussing the use of data collected from village health experts (ASHAs and Traditional Healers) and present the analytical sample size for the statistical analyses in the main text. Finally, we describe ethical procedures for the data collection.

1.1 Sample selection and sampling frame

From January 2020 to August 2022, we collected data in ten villages in the state of Meghalaya in the northeast of India. We studied three of the state's districts: West Khasi Hills, West Jaintia Hills, and South Garo Hills. These were among the most isolated areas of Meghalaya with high malaria incidence. For our study, we selected three villages in West Khasi Hills (labelled here as WK1, WK2, WK3), three in West Jaintia Hills (WJ1, WJ2, WJ3) and four in South Garo Hills (SG1, SG2, SG3, SG4). These villages were selected based on their manageable size (<500 eligible adults), their likely willingness to participate in the study (we avoided villages where previous epidemiological studies encountered high rate of refusals[1]), and their accessibility either by car or on foot.

We aimed to administer a structured face-to-face interview, based on a questionnaire developed specifically for this study, to every adult (of 18 years and older) in each of the ten villages. We used census data obtained from the register of the ASHA in every village to identify eligible individuals and construct our sampling frame. The ASHA's register contains an up-to-date record of every person living in a given village. It stores information by household, making it easy to identify who lived with whom at the time of the data collection. The format of the register is illustrated in **Figure S1**. Based on the ASHA records, we identified 2,239 eligible adults living in 952 households in the ten villages.

1.2 Total sample size and response rates

After interviewing every reachable adult, we were able to gather information from a total of 1,529 villagers (68.28% of eligible adults) residing in 764 households (80.25% of eligible households). **Table S1** provides details about the population of each village at the time of the data collection according to the ASHA's register. The table further presents individual- and household-level response rates. The population figures include minors and people who recently moved out of the village but were not yet removed from the register. The table highlights that the studied villages varied in size, between 151 and 1,206 individuals and 25 and 226 households. Response rates ranged from 53% to 88% of adults. We managed to interview at least 44 adults in every village. While individual response rates are between 50-70% in five villages, they are above 75% in the other five. At the same time, we achieved consistently high rates of participation on the household level, between 73% and 97%. This means that we have information about the characteristics and reported adoption of prevention behaviors from at least one adult in most of the households. We note that there is a substantial negative correlation between village size and response rate: the Pearson correlation coefficient between the number of eligible adults and the response rate is -0.53. This suggests that response rates tended to be higher in the smaller villages.

Table S2 provides an overview of non-respondents by reason for non-response in each village. For the 709 non-respondents, we recorded the reason why the interview could not be carried out. These included being temporarily out farming (113 adults), being temporarily out of the village (168), being unable to respond due to other reasons (148), having their door locked (17), or refusing to participate (247). There were 16 cases for which the reason for non-response was not known.

While non-respondents did not answer our questionnaire, they could still be mentioned by respondents as people they discuss health related issues with. This allows us to assess their position in the village network to some degree. While the average number of nominations for health-related discussions received by respondents is 1.85 (see Table S11), the same average is only 0.56 for non-respondents

(Table S12). This indicates that non-respondents were typically rather peripheral in the village social networks, talking to few people compared to villagers who were available and willing to be interviewed.

Further, the higher non-response rates in some of the villages are put in a different context by the reasons for non-response mentioned above. For example, WJ3 is the village where we achieved the lowest response rate (53%, which is over 10% points lower than the next lowest rate). In the same village, 50 of the 101 adults who did not participate in our study were reported being temporarily away or in fields (see Table S2), which means that they were effectively absent from the village community for a substantial amount of time. This together with the lower number of times non-respondents were mentioned as discussion partners increases the confidence in the validity of our results as network analyses tend to be less sensitive to missing data stemming from peripheral actors in the network[2].

1.3 Analytical sample size: use of data collected from village health experts

As we discussed in the main text, in the context of rural communities in Meghalaya there are two key individuals in almost every village whose roles directly relate to the provision of health-related information and treatments. The Accredited Social Health Activist (ASHA) represents the approach of modern medicine and the state-run health system in local communities. Their tasks include visiting revery household in the village regularly to keep demographic records, treating basic illness and injury with first aid, providing testing and medication for a variety of diseases including malaria, and distributing bed nets. The Traditional Healer typically advocates undocumented tribal treatments and prevention techniques largely based on medicinal plants. A survey[3] conducted in the Kasi and Garo Hills indicated that the estimated reported use of tribal medicine across rural households in Meghalaya was 79.1%. Tribal medicine was believed to be efficacious by 87.5% and was reported to be used for both minor ailments and major diseases. In the context of malaria prevention, both the ASHA and the Healer can be viewed as opinion leaders: they are health experts whose opinion, behavior, and influence may be substantially different from those of other villagers. Further, these experts, especially the ASHA, tend to be highly central actors in village social networks (see **Table S13**).

Identifying the ASHA of a village is straightforward as it is an official role that exactly one individual holds in each community. The role of the Healer is more informal and ambiguous. Since there is no official record of who is a Traditional Healer, we had to rely on participants' reports when identifying these individuals. When querying about health-related discussions, we asked them to report if someone they talked to was a Healer. This way, we could identify a single Healer per village in three villages (WK2, WK3, WJ2). Participants reported talking to two different Healers in WK1 and SG2, where we identified the one who was reported by more villagers as the Traditional Healer (for analytical clarity, we decided against using multiple Healers per village in our statistical models). The decision was clear in SG2, with 27 and 3 mentions of the two Healers, while it was closer in WK1 with 5 and 4 mentions. Village WJ1 was an exceptional case, as 13 villagers mentioned talking to the Healer of another village, WJ2, while no other Healer was mentioned. In this case, we decided that these two villages had the same Healer (who was only counted once as a participant, in their resident village of WJ2). Participants in the rest of the villages did not report talking to a Healer. Altogether, we were able to identify a Traditional Healer in six of the ten villages.

Due to their likely importance for the adoption of malaria prevention behaviors in their community, we interviewed the ASHA in all ten villages and the Traditional Healer in the five villages that had a Healer (with one Healer serving two villages). Reflecting their distinct roles in the villages, we removed these two types of actors as respondents from our analyses. This leaves us with an analytical sample size of 1,514 individuals. We report participant numbers that include ASHAs and Healers in the main text and this SI, because we utilize information collected from and about these individuals in the statistical analyses. Specifically, we keep details regarding a) their own prevention behaviors and b) if a villager reports talking to them about health-related issues. These data are used in the analyses explaining the behaviors and social networks of other villagers. In section 2, descriptive statistics are reported on the analytical sample, with ASHAs and Healers excluded, for closer alignment with the analyses presented in the main text and in section 4.

1.4 Research ethics

Interviews were bilingual, conducted in the appropriate local language (Khasi, Pnar, or Garo) and the official language of Meghalaya (English). Permission to conduct the study was granted by the head of each village. Further, respondents were briefed on the goals of the study and on privacy and data protection protocols. Based on this information, they were asked to sign an individual informed consent form before the interview if they were happy to participate. The name of the villages and personal identifiers of individual participants were altered or removed to preserve anonymity. Ethical approval for the study was obtained prior to any contact with the chosen villages from the Institutional Review Boards (IRBs) of Martin Luther Christian University, Shillong, Meghalaya, India and New York University, New York, NY, USA. For all fieldwork conducted during the COVID-19 pandemic, our team followed the general safety protocols recommended by the Indian government.

2 Questionnaire and variables

This section provides an overview of the questionnaire that was followed in the structured interviews and the distribution of the variables used in the analyses reported in the main text. The questions presented here were asked from every respondent in the total study sample (n=1,529), including village health experts. The descriptive statistics are reported for the analytical sample of 1,514 individuals, excluding health experts, to make them comparable with the statistical analyses reported in the main text. When calculating some of these descriptive statistics, we utilize information collected from and about the ASHAs and Traditional Healers.

2.1 Adoption of prevention behaviors

Table S3 lists the questions we asked participants about their adoption of malaria prevention behaviors (questions Q1-Q8). Questions and response options are presented in English translation, while originally they were asked in Khasi, Pnar, or Garo, depending on the tribe living in the village. In the analyses reported in the article, we did not utilize responses about the use of bednets without insecticide treatment (Q1), as nearly all interviewees said they used these. We also did not utilize responses about the use of mosquito mats, as none of the interviewees said they used these. For the present study, we recoded frequency of use of covering clothes (Q4) and insecticide cream (Q7) to use (“always”, “sometimes” responses) and non-use (“rarely”, “never” responses), to match the response categories of the other items.

Table S4 reports the village-level and total averages and standard deviations of the number of prevention behaviors adopted by households and individuals (maximum 8). We can see from the table that overall, both households and individuals report following three behaviors on average. Village-level averages range from 2.3 to 3.4. The figures suggest that more behaviors are adopted in villages from West Khasi Hills and West Jaintia Hills districts than in those from South Garo Hills. The sources of these differences are highlighted by **Table S5**, which reports the proportion of individuals adopting each of the eight behaviors in each village. The table shows that there is considerable variation in the level of adoption both between prevention behaviors and villages. LLINs, covering clothes, and coils are used by most participants overall. Boots, insecticide cream and burning materials are more frequently used in some villages but rarely in others. Gloves and vaporizers are uncommon in most villages. Villages in South Garo Hills show higher levels of use of LLINs and coils than those in the other districts. At the same time, people in these villages report using the other six techniques generally less frequently. These patterns may explain the lower number of prevention behaviors adopted in the villages from South Garo Hills in **Table S4**.

2.2 Individual characteristics

Table S6 lists the interview questions that were asked related to individuals’ socio-demographic characteristics and roles in their household (questions Q9-Q14). In the analyses reported in the main text,

age (Q10) is used as a continuous variable measured in years. We distinguished seven ordinal categories of level of education (Q13), with the highest category including the original answers of “graduate”, “postgraduate”, and “diploma”; responses in the “other” category were recoded to one of these seven education levels as appropriate based on the open-text responses. Further, we used a binary indicator variable for occupation (Q14), representing whether a participant worked in fields (responses “cultivator” and “agricultural laborer”) or not (all other responses). Gender (Q9), being the head of the household (Q11), and looking after sick family members (Q12) were analyzed as binary indicator variables.

Tables S7-S9 report the distribution of the six individual characteristics in each village and in total. **Table S7** shows that 58% of our respondents are female. The gender ratio of respondents varies between villages, with a higher proportion of female interviewees in the villages from West Jaintia Hills (64%, 64%, and 72%) than in the other districts (41%-57%). 51% of respondents reported that they were heads of their household, with notable variation between villages (36%-75%). Most respondents (88%) oversee caring for sick household members (between 67%-98% in villages). Overall, 68% of respondents reported an occupation that involves working in fields. This category was the mode, or most common value, in all but three villages (WK3, WJ3, SG3).

Table S8 highlights that the average age of respondents is 35.4 years. Averages and standard deviations of age appear similar across the villages, except for two in the South Garo Hills (SG1, SG3) district. The population of these villages is somewhat younger than those of the others (31.4 and 33.5 years on average). Based on **Table S9**, the level of education in the sample is low. In total, 42% of respondents reported no schooling and 29% had up to primary education. 13% had middle school and 14% some form of secondary education. Only 2% had a higher education degree. The distribution of education levels also varies between villages. Respondents from WK3, SG1, and SG3 appear to have somewhat higher levels of education than those in other villages, while those from WJ3 and SG2, somewhat lower.

2.3 Social networks and households

Health-related discussion networks were queried by two interview questions (Q15-Q16), as presented in **Table S10**. We use information about discussion ties within one’s village (Q15) to define the health-related discussion network of each village. As the contacts mentioned in this question were living in the same village of the respondent, we could match their names with our participant data and reconstruct village-level social networks. In our analyses, we focus on the network ties connecting our participants, disregarding the structure of connections to non-respondents. However, we do consider the number of non-respondents participants report talking to as a control variable in all analyses (see **Table S12** for descriptive information about this measure). The interview question about out-of-village ties (Q16) is the basis for the variables that involve network size outside of one’s village. We define this as the number of people mentioned by each respondent in question Q16 (see **Table S12**).

Table S11 reports descriptive statistics of the village-level discussion networks among participants constructed from responses to question Q15 in **Table S10**. The table presents the following statistics for each village: number of nodes (the number of participants who could name and be named as discussion partners), number of ties (the total number of discussion ties to other participants reported), and average degree (the average number of discussion ties to other participants reported). As we have seen in **Table S1**, there is variation between villages in the number of network nodes (respondents). Accordingly, the number of discussion ties reported also varies by village. At the same time, the average number of discussion ties reported by respondents is comparable across villages: average degree ranges between 1.13 and 3.45, with a mean of 1.85.

Table S12 presents village-level and overall average descriptive statistics for the number of reported discussion ties to non-respondents and people outside of one’s own village. Respondents typically name a low number of these contacts, less than one of each on average. However, we again see some variation between villages, with average network size ranging from 0.25 to 0.91 to non-respondents and from 0.17 to 2.01 to people outside the village.

Table S13 shows the proportion of individuals who report discussion ties to the ASHA or the Traditional Healer, as well as the percentage of households where at least one individual talks to the ASHA or the Healer. It is apparent that in the villages where a Healer was present, both individuals and households reported talking to these experts about health-related matters less frequently than to ASHAs. In total, 46% of respondents and 50% of households have a discussion tie to the village ASHA. The percentages vary widely between 20%-70% for individuals and 35%-90% for households. In contrast, only 7% of respondents and 11% of households report a discussion tie to the Healer, varying between 4%-24% for individuals and 5%-42% for households. Differences between villages are not trivially explained by district, the number of ties to the two health experts rather seems to be characteristic to the given village.

Combining network information (Q15) with data on prevention behaviors (Q1-Q8), we may compute the proportion of people each individual talks to who adopt each prevention behavior. This is one way how we may define network exposure to a given prevention behavior. To describe exposure within the household, we can assess if at least one other participating household member a respondent lives with reports adopting each prevention behavior. Both network and household exposure are defined slightly differently, as counts of network configurations, in the network models discussed in the main text (see section 4.2 for exact definitions). However, we believe the simple statistics presented here provide an intuitive understanding of the distribution of these key variables.

Table S14-15 report the average and median level of network exposure to each prevention behavior in each village and in total. The tables show that as the level of adoption varied between prevention behaviors and villages, so does the proportion of discussion partners of respondents who adopt a given behavior. Typically, most of individuals' reported contacts use LLINs (90% on average across all villages), covering clothes (63%), and coils (66%). Network exposure levels generally appear moderate and mixed between villages for boots (25%), insecticide cream (24%), and burning materials (23%). Network exposure to the use of gloves (5%) and vaporizers (13%) is low on average.

Table S16 presents the percentage of individuals who are exposed to a prevention behavior within their households in each village and in total. While the measure of exposure is different here, we see similar patterns as in case of network exposure. LLINs (79% on average across all villages), covering clothes (59%), and coils (61%) tend to be used in most households. There is generally moderate and mixed use of boots (25%), insecticide cream (16%), and burning materials (25%). The use of gloves (6%) and vaporizers (9%) is low on average on the household level.

3 Visualization of village-level behavior adoption and discussion networks

Figures S2-S6 visualize the multilevel network system of each village as a combination of two networks. The first is a one mode network, connecting villagers to each other by their reported discussion ties. The second network connects villagers to any of the eight prevention behaviors that they pursue. In each figure, participants are represented by circle nodes and prevention behaviors by square nodes. Thick red edges connect two villagers if at least one of them reported talking to the other about health-related matters (we consider tie directions in the statistical analyses). Thinner edges of various colors connect villagers to the prevention behaviors they adopt (edges are colored by the prevention behavior they are connected to). The figures in this section were produced using the 'ggraph' package (v2.1.0)[4] with the multilevel network layout implemented in the 'graphlayouts' package (v1.0.0)[5] in R.

The network graphs provide a glimpse at the structure of discussion ties and the patterns of prevention behaviors in the villages. The discussion networks appear sparse in all villages, in line with the low average degrees of around 2 reported in **Table S11**. Most village networks have one large component of connected nodes and several smaller ones. The exceptions are WK3 (**Figure S3**), where the entire network save for one participant forms a single component, and SG3-SG4 (**Figure S6**), which consist of a number of smaller ones. All village networks contain sets of actors within components who appear quite densely connected (in clusters). In terms of the number of ties each actor has (degree), almost all

networks are decentralized, except for SG2 (**Figure S5**), where one participant is clearly more central than the rest.

The graphs further provide some additional insight into prevention behaviors beyond the levels of adoption reported in **Table S5**. The common thicker "bundles" of edges linked to the eight nodes representing prevention behaviors signal higher levels of adoption in specific regions of the discussion network. For example, in case of boots in WK2 (**Figure S2**), we can see edges spanning out to three sets of participants. Each set represents pairs of individuals who are connected in the discussion network. In this sense, edge thickness in these graphs may be interpreted as a sign of network autocorrelation: similarity in adopted prevention behaviors among those individuals who are connected by discussion ties. Naturally, given the number of nodes and the complexity of discussion networks, these patterns cannot be fully represented in 2D graphs. However, relations between behaviors and discussion ties are explicitly quantified and tested in the stochastic actor-oriented models reported in the main text and described in section 4 below.

4 Details of the analyses using stochastic actor-oriented models (SAOMs)

This section presents the details of the SAOM analyses reported in the main text (**Figures 4-6**). We first describe our modelling approach, with reference to the SAOM framework (section 4.1). We then discuss model specification and provide definitions for the effects (explanatory variables) we use in the model (section 4.2). Further, we present village-level SAOM results (section 4.3) and full results for the SAOM meta-analysis reported in the main text (section 4.4); the latter were used to create **Figures 4-5** in the main text. We continue by providing details for the comparison of the fit of different nested model specifications in village-level models (section 4.5); the results of this step provide the basis for **Figure 6** in the main text. Lastly, we present robustness checks for the rate parameters of the stationary SAOMs (section 4.6).

4.1 Overview of modelling approach

In our village-level stationary SAOMs, we jointly model two networks: one representing discussion ties between villagers and one representing the adoption of different prevention behaviors by villagers. We used the RSiena package in R[6] to fit the village-level models, and the metafor package[7] to perform the meta-analyses of the results.

The main network of interest is a two-mode network connecting villagers to prevention behaviors. The presence of a tie in this network means that a given villager reported pursuing the behavior in question, whereas a tie's absence means they did not. This way, we do not distinguish between prevention behaviors in the model. This is not a shortcoming of the SAOM framework: we could study processes specific to certain behaviors using node covariates. However, the advantage of assuming that social processes are comparable across behavior is more statistical power.

Our second modelled network is the one-mode discussion network about health-related issues connecting villagers. The presence of a tie here means that a given villager reported talking to the other villager in question; tie absence means a lack of such a mention. This network is directed as it is possible that either or both members of a pair (dyad) of villagers recalls or finds a discussion relevant to mention.

The two-mode and one-mode networks jointly define a multilevel network of discussions and behavior adoption in each village. These networks are visualized as graphs in section 4, **Figures S2-S6**. We apply the SAOM to explore which network mechanisms may keep the structure of these multilevel networks stable over a short period of time. While changes in the discussion network are explicitly modelled in our approach, we focus on interpreting results about the adoption of behaviors as these are focal to our research question. Results for the discussion network are viewed as "control" variables, which allow better estimation of parameters explaining the adoption of behaviors. We ensured nonetheless that the final models provided a reasonable representation (good fit) of the discussion networks as well.

4.2 Model specification and definition of effects

The specification of effects, the term used for explanatory variables in the SAOM, in the fitted models was determined by our research question (effects of various individual and social factors on the prevention behaviors), earlier empirical findings (role of individual characteristics, health experts, and social networks), and established practices in the field of dynamic network modelling with SAOMs[6,8]. **Tables S17** and **S18** list the effects that were included in the full models, for the behavior adoption and the discussion networks respectively, along with their "short names" based on which they can be exactly identified in section 12 of the RSiena Manual[6]. Where relevant, linear effects of network statistics (as opposed weighted sums) were used by setting appropriate effect parameters to 1 in RSiena. Only key effects were presented in **Figures 4-5** in the main text, but models were estimated will all effects listed here included. Similarly, all effects were used in the model comparisons of **Figure 6** of the main text.

4.3 Village-level SAOM results with score-type tests

We first attempted to fit a model with the above specification in each of the ten villages. In cases when a model did not converge or was not precisely estimable[9], we "fixed" parameters for effects with problematic estimates. That is, we constrained them to a constant value prior to estimation. Problematic effects were identified by unusually large standard errors (and sometimes estimates too). We tested fixed parameters using score-type tests as suggested in section 8.2 of the RSiena Manual[6]. Effect fixing followed a stepwise approach, fixing one additional effect at a time, until the model converged. Initially, each affected parameter was fixed to the value 0; this value was then adjusted if the score-type tests were statistically significant at the 5% level. In the models reported in the main text and this document, all score-type tests are non-significant at a 5% level, suggesting that the chosen constraints were adequate. The list of constrained effects and detailed score-type test results are reported in **Tables S19-S20**.

Tables S21 and **S22** present the results of ten village-level SAOMs, estimated with identical model specifications (except for constrained effects as discussed above). All of the presented models converged according to the criteria that all effect-wise convergence t-statistics are smaller than 0.1 in absolute value and the overall maximum convergence ratio is smaller than 0.25[6]. The models were estimated using rate parameters of 3 for both networks – this choice is further discussed and scrutinized in section 4.6.

Table S21 shows that there are only two effects explaining the adoption of behaviors that are statistically significant in several villages (apart from the intercept, density – effect 1 in the table). One is trivial: the prevalence of behaviors (inPop – effect 3) effect reflects the tendency that some behaviors are adopted by many participants, while others are adopted by only a few. The other relevant effect is network exposure (to – effect 22), which is positive in every case, suggesting that discussion ties increase the likelihood of adopting the same prevention behaviors.

Table S22 shows a higher number of statistically significant estimates from factors explaining discussion ties. Some network structural effects are found consistently in almost all of the villages: discussion ties are more likely to be reported if they are mutual (recip – effect 2), ties are more likely to appear in clusters of closed triads (transTrip – effect 3), the effects of mutuality and clustering are subadditive (transRecTrip – effect 4), and those with more ties sent are less likely to send additional ties (outAct – effect 6). Further, those who are female (altX – effect 14), older (altX – effect 26), or of similar age (simX – effect 27) are more likely to be reported as discussion partners. Notably, there is strong evidence that those in the same household are more likely to discuss health-related matters than those who live in different households (sameX – effect 39). At the same time, we see a statistically significant effect from adopting the same behaviors on discussion ties in one village only (from – effect 40).

4.4 Full SAOM meta-analysis results

Tables S23 and **S24** present the full results of the effect-wise meta-analyses of village-level SAOMs, including heterogeneity statistics. In both tables, we report the estimated mean of the village-level parameters (μ), its standard error (se), the p-value of the test μ=0 (p), the estimated between-village standard deviation of parameters (τ), Cochran's Q that reflects the variability of village-level parameters

around the fixed-effects estimate of their mean (Q), the p-value of the test Q=0 (Qp), and the number of village-level models used in each parameter-wise meta-analysis (n). We note that n may be different across model terms because some parameters had to be constrained in some of the villages to make all models estimable. This is explained in detail in section 4.3 above. Since constrained parameters were not estimated, they are not included in the meta-analyses reported here and in the main text.

Based on **Table S23**, we find evidence for a baseline tendency to adopt few prevention behaviors (density – effect 1 in the table) and for certain behaviors being more prevalent than others (inPop – effect 3). Talking to the ASHA increases the probability of behavior adoption in general (egoX, talks to the ASHA – effect 11); that is, those who talk to the ASHA tend to adopt a larger number of behaviors than those who do not. The size of individuals' discussion networks seems to matter for prevention behavior in two ways: naming more participants as contacts within one's village on average decreases the probability of adopting behaviors (outActIntn – effect 17), while more contacts with non-respondents (egoX, outdegree to non-respondents – effect 19) and people outside of the village (egoX, outdegree out of village – effect 20) increase the likelihood of behavior adoption. Further, we do not find a significant tendency for household members to pursue the same prevention behaviors (sameXCycle4 – effect 21). As we see in the main text, this does not mean that households do not matter. We also consider the effect of living in the same household on discussion ties in the model, as explained below. Finally, individuals tend to adopt the same behaviors as the discussion contacts they report do (to – effect 22). We note that this result should be interpreted considering that we control for the effect of similarity in behaviors on discussion ties in the model, as explained below.

Regarding the heterogeneity of village-level estimates, **Table S23** suggests considerable variance in structural effects (effects 1-4), the effect of education on the number of behaviors adopted (effect 10), and the effect of discussion contacts' behaviors on one's own behaviors (effect 22). Regarding the last finding, it should be noted that even though the parameter of the "to" effect may vary between villages, the village-level SAOM results presented in **Table S21** show that this effect was estimated to be positive in all villages and it was statistically significant at the 5% level in 6 out of 10 villages. This provides strong evidence for an overall positive estimate for this model effect, despite the observed heterogeneity.

Table S24 provides details about the meta-analysis results for SAOM effects explaining the presence of discussion ties between villagers. These parameters were simultaneously estimated in the SAOM with those explaining behavior adoption above. The meta-estimates suggest a baseline tendency for reporting a low number of discussion partners (density – effect 1), a tendency for discussion ties being reciprocated (recip – effect 2), for clustering in the discussion network (transTrip – effect 3), for a sub-additive interaction between effects of reciprocation and clustering (transRecTrip – effect 4), and against high numbers of ties sent (outAct – effect 6). There is also a positive effect of discussion ties outside one's village on reporting contacts within the village (egoX, outdegree outside of village – effect 7).

Further, it can be seen in **Table S24** that some individual characteristics appear to shape the discussion networks similarly across villages: female participants are more likely to receive discussion ties (altX, female – effect 14) and report discussions with other female villagers (sameX, female – effect 15); heads of households are more likely to receive ties as well (altX, head of household – effect 17). Those in care of sick people in their households are more likely on average to report health-related discussions (egoX, in care of sick – effect 19) and to receive discussion ties (altX, in care of sick – effect 20). Participants are more likely to report discussion ties to older villagers (altX, age – effect 26) and to those who are of similar age as themselves (simX, age – effect 27).

Effects related to village health experts in **Table S24** highlight that villagers are more likely to report discussion ties with each other if both of them talk to the Traditional Healer about health-related issues (sameX, talks to Healer – effect 36); we note that this is based on results from only four villages due to the absence of a Healer in four villages and the low number of discussion ties reported with the Healer in two villages. Those in the same household are more likely to report discussion ties with each other than with members of other households (sameX, household – effect 39).

There is evidence in **Table S24** for two negative effects from the number of behaviors pursued: those who adopt more behaviors tend to report (outActIntn – effect 37) and be reported by (outPopIntn –

effect 38) fewer villagers as discussion contacts. Lastly, we find a higher tendency for those who adopt the same behaviors to be connected in the discussion network than those who adopt different ones (from – effect 40); we note that this effect is taken into account by model design when the effect of discussion ties on adopting the same behaviors is estimated (the "to" effect in **Table S23**).

Regarding the heterogeneity of village-level estimates, **Table S24** suggests considerable variance in some structural effects (effects 3, 4, 6), the effect of being head of the household on the tendency to receive discussion ties (effects 17), the effect of talking to the ASHA on sending ties (effect 31), and the effect of living in the same household on being connected in the discussion network (effect 39).

4.5 Assessment of the goodness of fit of village-level SAOMs

We assess the goodness of fit (GoF) of the SAOMs presented above in each village by a number of network statistics using the sienaGOF function in the RSiena package[10] in R. We use nine sets of statistics for these tests:

- (1-2) the outdegree and indegree distributions in the behavior adoption network,
- (3-4) the distribution of mixed triads including adoption and discussion ties according to the full mixed triad census[11] and a reduced set of triads representing similarity of behavior adoption in pairs of villagers (the 22, 21, 20, 02, 01, and 00 triads from[11]),
- (5-9) the outdegree, indegree, and geodesic distance distributions, triad census, and clique census (up to 5-cliques) of the discussion network.

Table S25 presents the p-values for Mahalanobis distances[12] of the observed statistics from the same statistics obtained from 5,000 networks simulated based on the fitted village-level models. In all of the villages, the models adequately fit several structural properties of both the behavior adoption and discussion network, as well as their mixed triadic configurations based on guidelines given in the RSiena Manual[6]. All p-values are larger than 0 and many are close to 1, suggesting an excellent fit of the models on several structural properties of the two networks. Most importantly for our study, the fit of models on the mixed triad census (line 3 in the table) is good in most villages. P-values in WJ1, WJ3, and SG4 are lower, but they are not exactly zero, which signals acceptable fit in these cases as well.

Table S26 presents and overview of model fit in the GoF comparisons using the mixed triad census for each village (line 3 in **Table S25**). This table provides the data for the results presented in **Figure 6** of the main text. The seven model specifications compared are described in the main text, in section 1, and are presented in **Tables S17-S18**. The structural model is a sensible baseline model that assumes that both networks are shaped and, in our case, kept stable, by the existing patterns of their ties. Overall, **Table S26** shows that model fit on the mixed triad census is rather poor in case of specifications that include effects of network structure, individual characteristics, ties to health experts, and network size (lines 1-4), with average p-values between 0.08 and 0.20. There is a large improvement in fit once household exposure or network exposure is taken into account (lines 5-6), on average to 0.42 and 0.45, respectively. Including both exposure effects in the models (line 7) further improves fit, with the average p-value increasing to 0.56. Crucially, only this full model achieves adequate fit on the mixed triad census in all villages, as marked by all p-values being larger then exactly 0[6]. The model specification with the best fit is the full model which provides the results for **Figures 4-5** of the main text and the above sections.

For a statistical assessment of the differences in goodness of fit between models, **Table S27** reports results of t-test for the differences in average GoF p-values for different SAOM specifications. This table also contributes to **Figure 6** in the main article. The first line contains the result of a one-sample t-test of the mean p-value of the baseline models across villages. The other lines report paired sample t-tests comparing the mean p-values of the model specifications highlighted in the table. The tests demonstrate that including network exposure and/or household exposure in the models leads to a significant improvement of fit over the network size model. At the same time, including both effects only leads to an improvement that is significant at 10% over the household exposure (p=0.060) and the network exposure model (p=0.087). However, we pointed out above that both effects are necessary to achieve an adequate fit in every village.

4.6 Robustness checks with different SAOM rate parameters

In SAOMs, the rate parameter governs the frequency of change in a modelled (dependent) network within the simulation model. In longitudinal applications of the model, rate parameters are estimated based on the data. In stationary SAOMs, such as in our models, this is not possible as we do not observe network changes in our cross-sectional dataset. In this case, rate parameters need to be set by the researcher arbitrarily and need to be assessed by robustness checks[13].

The rate parameters in the models we report in the main text and in this section are set to 3 for both networks. While this number may seem low compared to the few existing examples[13,14], it is reasonable in our context. The overall average number of ties sent by villagers in the discussion network is around 2, and 3 in the use network. This means that by setting the rates to 3, an average villager can expect to get a chance to change as many as all of their outgoing ties in the simulation model component of our SAOMs. Considering that the number of second-mode nodes in the measure use network is only 8, this means that the average number of change opportunities per person equal more than third of all measures that villagers may use. Altogether, this suggests that rates of 3 represent a reasonable "short-term" timescale in our networks, in which we may expect the multilevel network system to be in a stationary state, in line with the model assumptions[13].

Nonetheless, we experimented with higher rate parameters as robustness checks for our results. **Table S28** shows a comparison of SAOM meta-analysis estimates from three models with different rate parameters: 3-3 (the results reported in the main text and in this section so far), 5-5, and 8-8. In the last case, models in more than half of the villages did not converge. The meta-analytic results in this case are based on only a couple of villages and, thus, only serve for comparison of effect sizes and directions. Rates higher than 8 resulted in even more convergence problems, and so such models were not explored further. Overall, the table suggests that our key results are robust to the choice of rate parameters in this range: estimates have the same sign and similar size across the models and there is only little change in which effects are found significant at the 5% level. The effect network exposure (to – effect 22) remains significant and comparable in size in all three models. The effect of household exposure (sameXCycle4 – effect 21) is of similar size and non-significant across the models. At the same time, household-related mechanisms were still important for explaining discussion ties (sameX – effect 39) and achieving a good model fit. This pattern of findings motivates our conclusions in the main text regarding network exposure as a key factor explaining prevention behaviors and households as foci for health-related discussions in the studied villages.

5 Figures

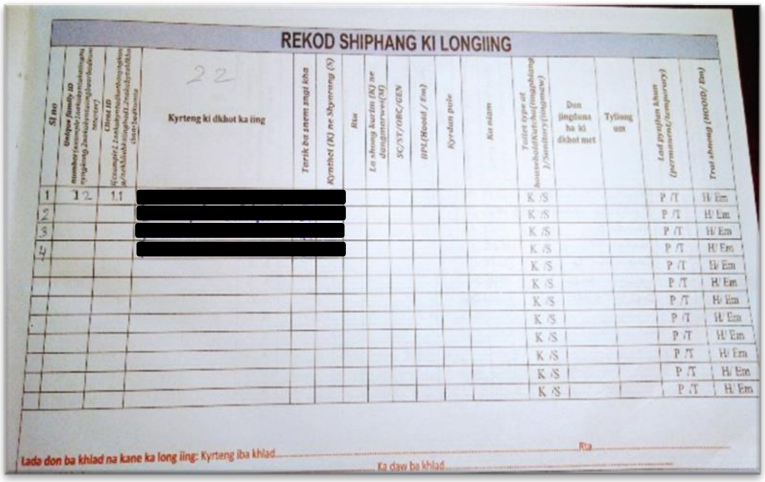

Fig. S1. An example page from the ASHA's register in one of the studied villages. *The document contains an up-to-date record of who lives in the given village. Each page lists the members of a single household. We used these records to compile the sampling frame for our study. Individuals' names on the page were redacted for anonymity.*

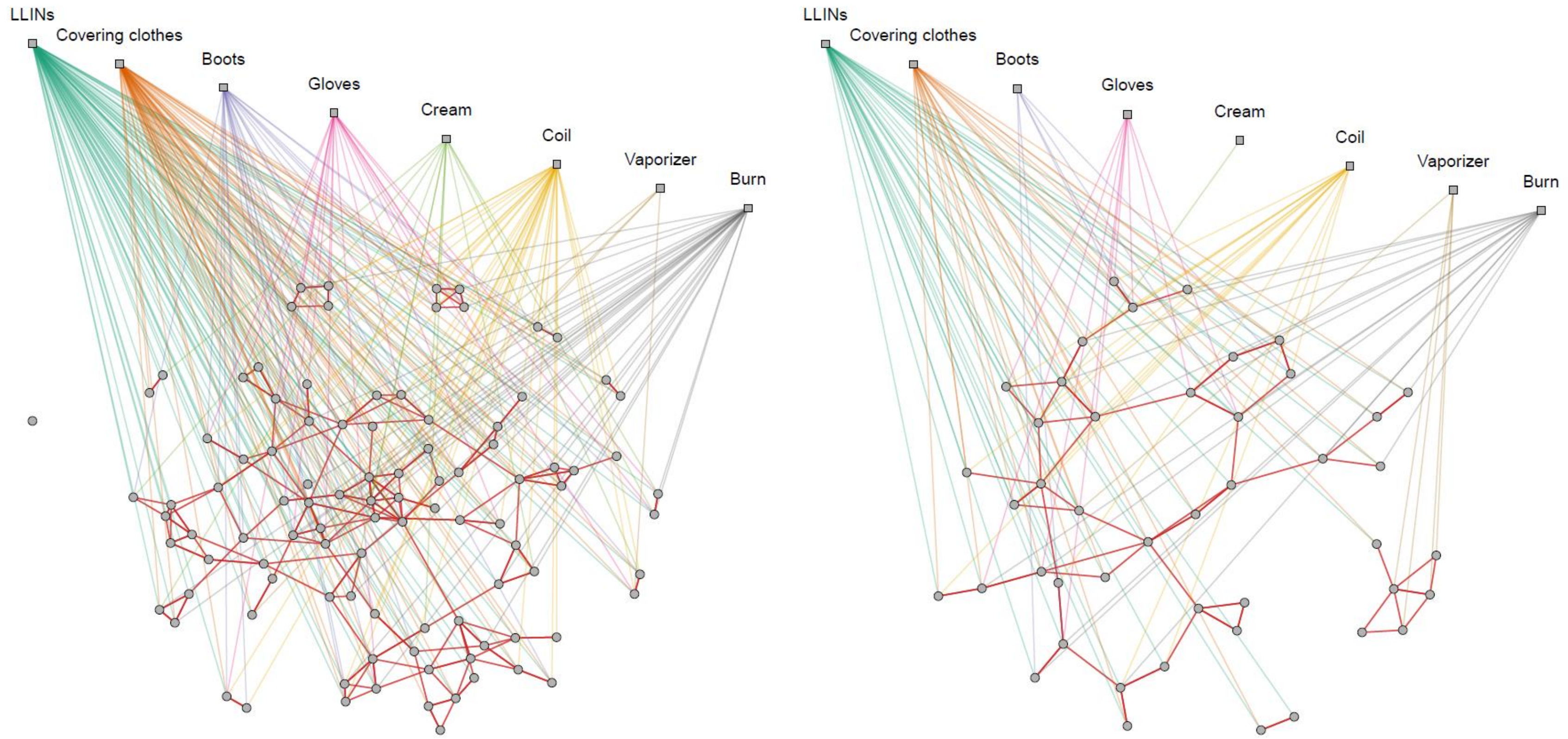

Fig. S2. Visualization of the multilevel network of discussion ties and prevention behaviors in WK1 (left) and WK2 (right). *Circle nodes: participants, square nodes: prevention behaviors, thick red edges: discussion ties reported by at least one of the two involved participants, thin edges: measure use with edge color specific to prevention behaviors; multilevel graph layout by the 'graphlayouts' package in R*[5].

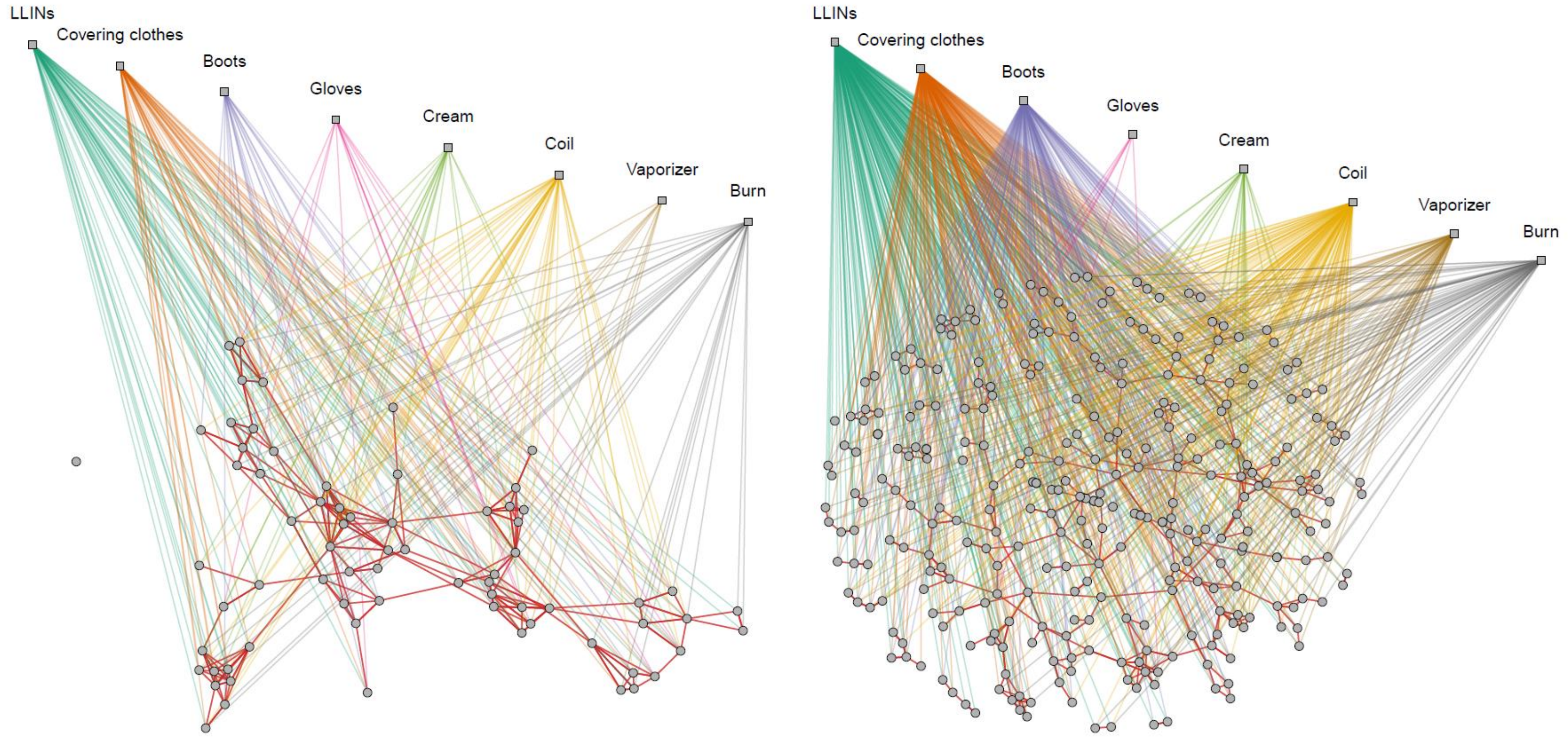

Fig. S3 Visualization of the multilevel network of discussion ties and prevention behaviors in WK3 (top) and WJ1 (bottom). *Circle nodes: participants, square nodes: prevention behaviors, thick red edges: discussion ties reported by at least one of the two involved participants, thin edges: measure use with edge color specific to prevention behaviors; multilevel graph layout by the 'graphlayouts' package in R*[5].

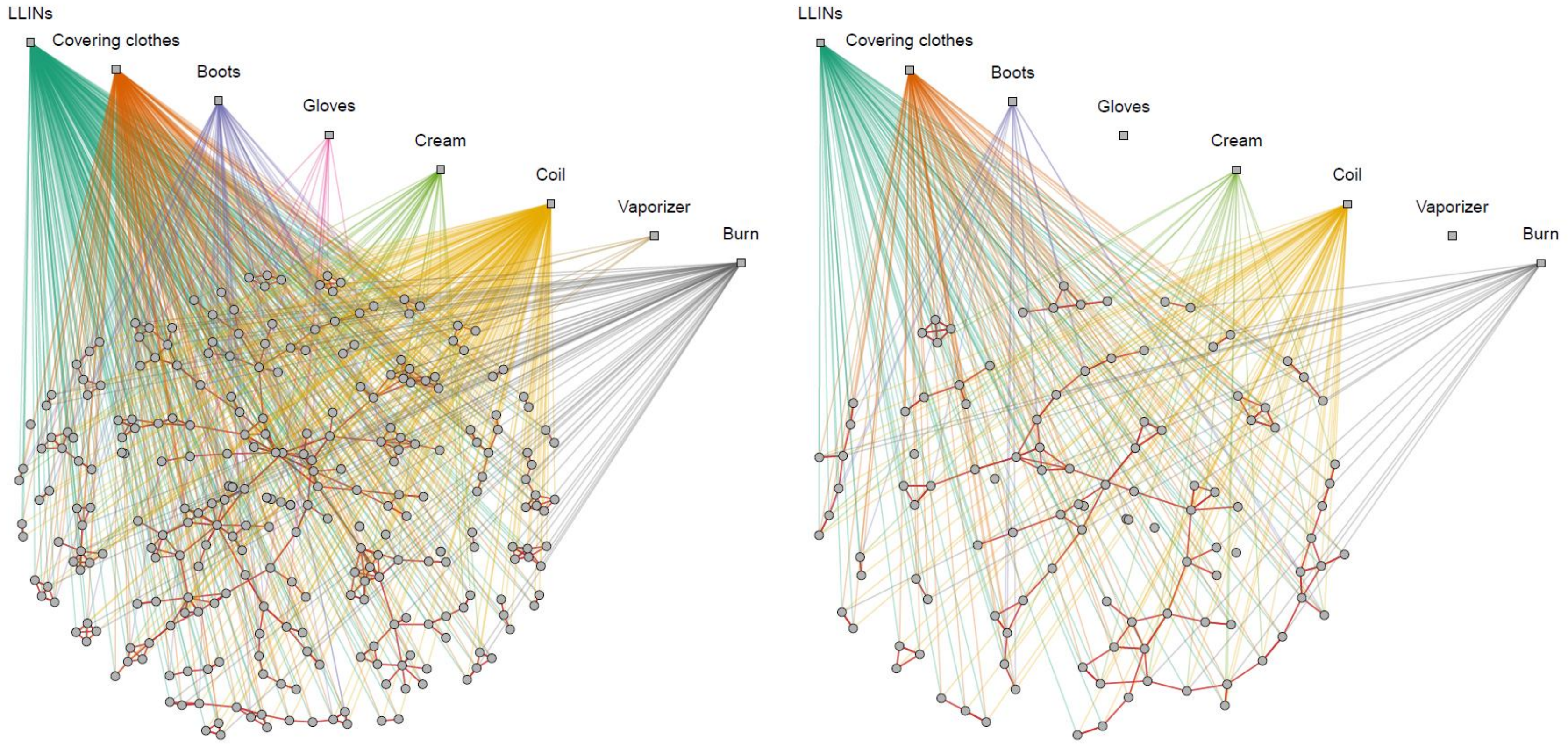

Fig. S4. Visualization of the multilevel network of discussion ties and prevention behaviors in WJ2 (top) and WJ3 (bottom). *Circle nodes: participants, square nodes: prevention behaviors, thick red edges: discussion ties reported by at least one of the two involved participants, thin edges: measure use with edge color specific to prevention behaviors; multilevel graph layout by the 'graphlayouts' package in R*[5].

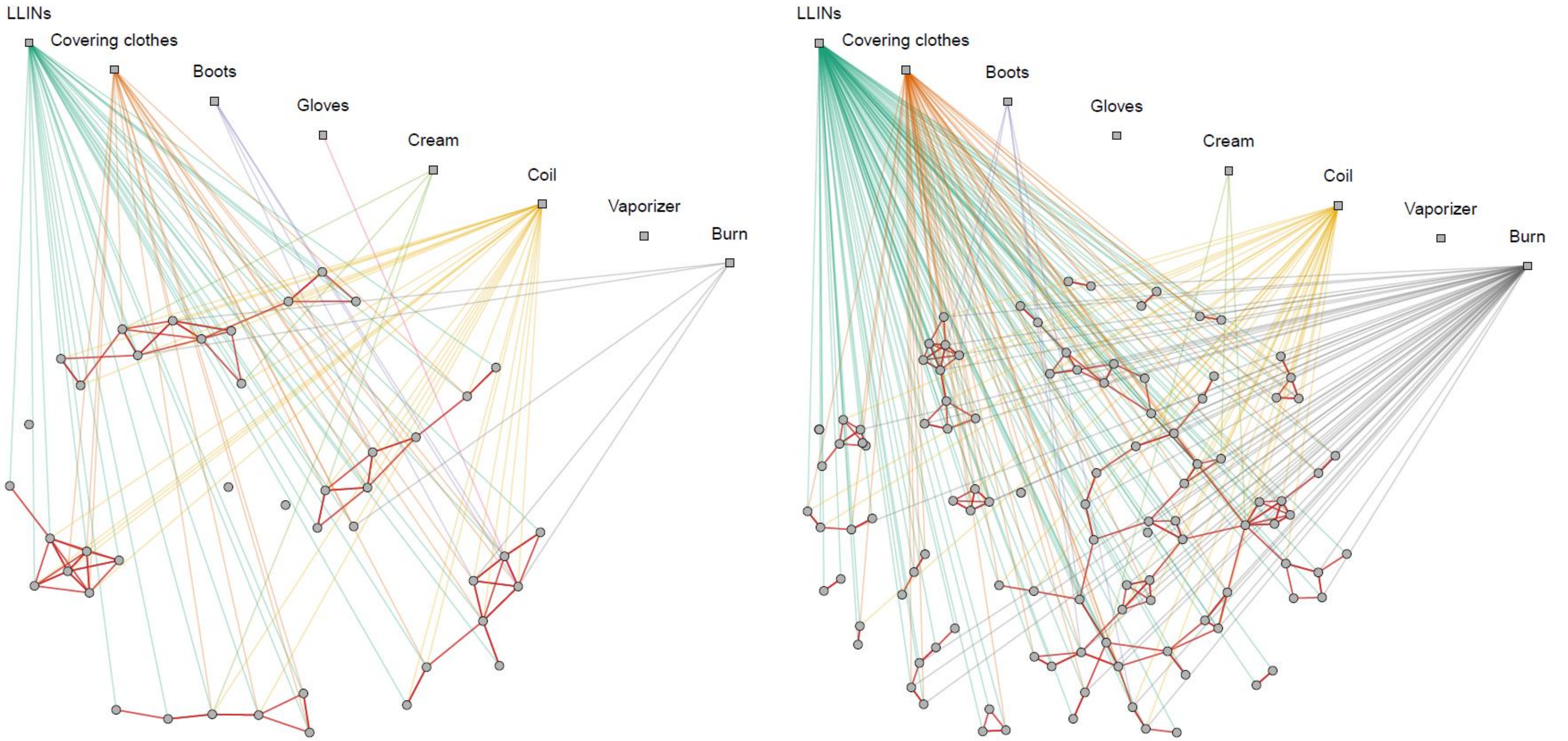

Fig. S5. Visualization of the multilevel network of discussion ties and prevention behaviors in SG1 (top) and SG2 (bottom). *Circle nodes: participants, square nodes: prevention behaviors, thick red edges: discussion ties reported by at least one of the two involved participants, thin edges: measure use with edge color specific to prevention behaviors; multilevel graph layout by the 'graphlayouts' package in R*[5].

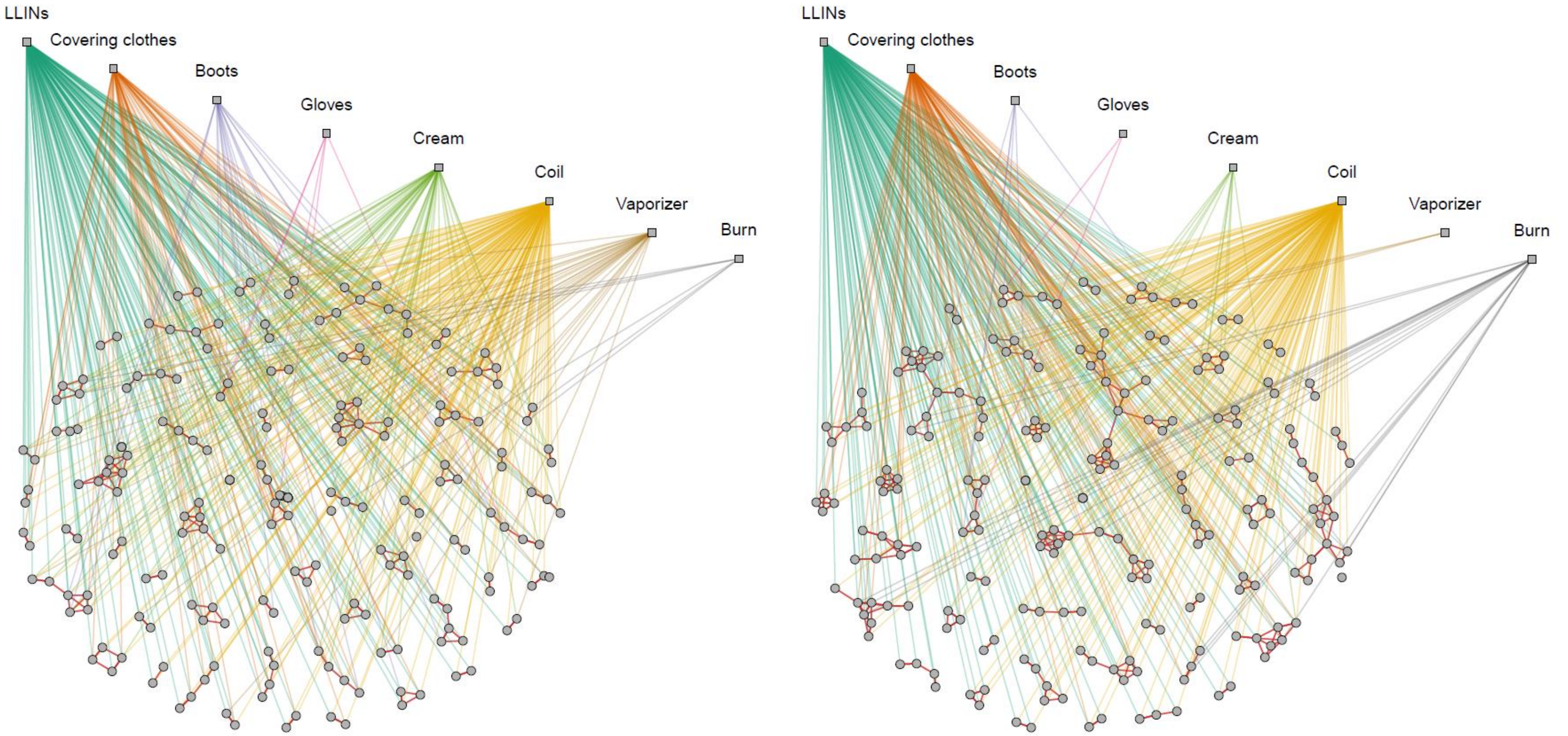

Fig. S6. Visualization of the multilevel network of discussion ties and prevention behaviors in SG3 (top) and SG4 (bottom). *Circle nodes: participants, square nodes: prevention behaviors, thick red edges: discussion ties reported by at least one of the two involved participants, thin edges: measure use with edge color specific to prevention behaviors; multilevel graph layout by the 'graphlayouts' package in R*[5].

6 Tables

Table S1. Population size and response rates by village and in total.

District	West Khasi Hills			West Jaintia Hills			South Garo Hills				Total
Village	WK1	WK2	WK3	WJ1	WJ2	WJ3	SG1	SG2	SG3	SG4	
Population	255	151	187	1206	1066	546	163	328	644	589	5135
Eligible adults	130	54	83	484	458	216	68	152	315	278	2238
Adults interviewed	102	47	73	316	291	115	44	117	212	212	1529
% eligible adults interviewed	78%	87%	88%	65%	64%	53%	65%	77%	68%	76%	68%
Eligible households	69	25	31	226	190	98	30	56	106	121	952
Households interviewed	52	21	30	175	151	78	22	50	94	91	764
% eligible households interviewed	75%	84%	97%	77%	79%	80%	73%	89%	89%	75%	80%

Table S2. Number of non-respondents by reason of non-response in each village and in total.

District	West Khasi Hills			West Jaintia Hills			South Garo Hills				Total
Village	WK1	WK2	WK3	WJ1	WJ2	WJ3	SG1	SG2	SG3	SG4	
Eligible adults	130	55	83	485	460	216	68	152	312	278	2239
Temporarily in fields	6	0	0	37	27	32	4	7	0	0	113
Temporarily away	13	5	3	33	31	18	6	10	35	14	168
Unable to respond	4	2	1	28	21	14	11	9	23	35	148
Door locked	0	0	0	6	4	2	0	0	5	0	17
Refused to respond	3	0	0	64	84	35	3	5	36	17	247
Unknown reason	2	0	6	0	0	0	0	4	4	0	16
Total non-respondents	28	7	10	168	167	101	24	35	103	66	709

Table S3. Interview questions about the adoption of malaria prevention behaviors (Q1-Q8). *Items and response options are described in English translation; originally, they were asked in Khasi, Pnar, or Garo, depending on the tribe of the village*.

No.	Question (in English)	Response options
Q1	Do you use a net at night?	Yes [ ] No [ ] *(If no, go to Q4)*
Q2	If yes, is the net treated with insecticide (LLINS)?	Yes [ ] No [ ] Don’t know [ ]
Q3	If you do not use a net, what is the reason?	*[free response]*
Q4	Do you cover your arms and legs during evening and early morning to prevent mosquito bites?	Always [ ] Sometimes [ ] Rarely [ ] Never [ ]
Q5	[If they work in fields:] Do you use boots when you go farming?	Yes [ ] No [ ] Don’t know [ ]
Q6	[If they work in fields:] Do you use gloves when you go farming?	Yes [ ] No [ ] Don’t know [ ]
Q7	Do you use insecticide cream to prevent mosquito bites?	Always [ ] Sometimes [ ] Rarely [ ] Never [ ]
Q8	Do you use any of the following to prevent mosquito bites? *(Tick all that apply)*	Use coils [ ] Use vaporizer [ ] Mosquito mats [ ] Burn material (like neem leaves, cow dung) [ ] Other (specify) [ ]

Table S4. Number of prevention behaviors adopted by households and individuals in each village and in total. *Avg. = average; S.d. = standard deviation.*

Village	Avg. no. of behaviors by households	S.d. no. of behaviors by households	Avg. no. of behaviors by individuals	S.d. no. of behaviors by individuals
WK1	2.7	1.2	2.8	1.4
WK2	2.6	1.3	2.5	1.4
WK3	3.3	1.0	3.4	1.2
WJ1	3.3	1.0	3.3	1.1
WJ2	3.1	0.9	3.2	1.0
WJ3	3.2	1.0	3.1	1.1
SG1	2.5	0.8	2.3	1.1
SG2	2.4	0.9	2.4	1.1
SG3	2.7	0.7	2.7	0.9
SG4	2.5	0.8	2.5	0.9
Total	2.9	1.0	2.9	1.1

Table S5. Proportion of individuals adopting the studied prevention behaviors in each village and in total. *Percentages in valid cases, excluding missings, are reported in rows 1-11; missing ratios of all cases is reported row 12. Cell color indicates level of use: higher levels marked by darker green.*

Village	LLINS	Covering clothes	Boots	Gloves	Insecticide cream	Coils	Vaporisers	Burning materials
WK1	80%	64%	26%	20%	13%	38%	4%	30%
WK2	82%	53%	13%	20%	2%	36%	11%	36%
WK3	89%	72%	30%	17%	24%	58%	13%	38%
WJ1	98%	77%	49%	3%	9%	52%	22%	24%
WJ2	99%	69%	24%	4%	14%	78%	3%	25%
WJ3	91%	80%	18%	0%	22%	75%	0%	23%
SG1	88%	47%	9%	2%	9%	67%	0%	12%
SG2	100%	50%	4%	0%	3%	33%	0%	49%
SG3	95%	36%	12%	2%	25%	86%	15%	3%
SG4	94%	48%	3%	1%	5%	85%	1%	9%
Total	95%	61%	22%	4%	13%	66%	9%	22%
Missing	0%	0%	0%	0%	0%	0%	0%	0%

Table S6. Interview questions about individual characteristics (Q9-Q14). *Items and response options are described in English translation; originally, they were asked in Khasi, Pnar, or Garo, depending on the tribe of the village.*

No.	Question (in English)	Response options	
Q9	Gender	Male [ ]	Female [ ]
Q10	Age	*[age recorded in years]*	
Q11	Are you the head of the household?	Yes [ ]	No [ ]
Q12	Are you in charge of looking after other members of the family when they are sick?	Yes [ ]	No [ ]
Q13	Highest level of education	No schooling [ ]<br>Primary (1-5) [ ]<br>Secondary (X) [ ]<br>Graduate [ ]<br>Diploma [ ]	Below primary [ ]<br>Middle (6-8) [ ]<br>Higher Secondary (XII) [ ]<br>Postgraduate [ ]<br>Other (specify) [ ]
Q14	Usual occupation	Cultivator (independent farmer) [ ]<br>Agricultural labourer [ ]<br>Salaried service [ ]<br>Housewife [ ]<br>Child, not in school [ ]<br>Other (specify) [ ]	 Daily wage/labour [ ]<br>Self-employed/trade [ ]<br>Student [ ]<br>None [ ]

Table S7. Percentage of respondents who are female, heads of households, carers for a sick person, or working in fields in each village and in total. *Percentages within valid cases, excluding missing, are reported in rows 1-11; missing ratio of all cases is reported row 12. Cell color indicates proportion of respondents: larger numbers marked by darker green.*

Village	Female	Head of household	Carer for a sick person	Works in fields
WK1	57%	47%	77%	85%
WK2	49%	56%	67%	73%
WK3	54%	36%	67%	39%
WJ1	64%	37%	86%	80%
WJ2	64%	43%	89%	58%
WJ3	72%	39%	87%	46%
SG1	51%	67%	88%	85%
SG2	41%	63%	94%	87%
SG3	51%	59%	96%	48%
SG4	52%	75%	98%	78%
Total	58%	51%	88%	68%
Missing	0%	0%	0%	1%

Table S8. Average and standard deviation of age in each village and in total. *Statistics calculated for valid cases, excluding missing, are reported in columns 1-2; missing ratio of all cases is reported column 3; Avg. = average; S.d. = standard deviation.*

Village	Avg. age	S.d. age	Missing
WK1	35.9	13.8	0.00%
WK2	35.0	14.0	0.00%
WK3	36.8	14.7	0.47%
WJ1	35.9	12.5	0.47%
WJ2	36.2	13.3	0.00%
WJ3	34.3	11.7	0.00%
SG1	31.4	11.6	0.88%
SG2	35.4	12.6	0.00%
SG3	33.5	10.3	0.00%
SG4	36.2	13.1	1.41%
Total	35.4	12.7	0.26%

Table S9. Distribution of highest level of education across the seven education categories in each village and in total. *Percentages within valid cases, excluding missing, are reported in columns 1-7; missing ratio of all cases is reported column 8. Cell color indicates proportion of respondents: larger numbers marked by darker green.*

Village	No schooling	Below primary	Primary	Middle	Secondary	Higher secondary	Graduate and above	Missing
WK1	42%	19%	27%	11%	0%	0%	1%	0.00%
WK2	40%	16%	27%	2%	2%	7%	7%	0.00%
WK3	13%	4%	23%	19%	13%	17%	11%	1.41%
WJ1	46%	11%	30%	7%	3%	2%	0%	0.00%
WJ2	40%	7%	28%	10%	7%	6%	1%	0.00%
WJ3	58%	13%	15%	4%	7%	2%	1%	0.88%
SG1	33%	9%	12%	33%	14%	0%	0%	0.00%
SG2	69%	5%	5%	11%	10%	0%	0%	0.00%
SG3	27%	8%	9%	25%	22%	6%	2%	0.47%
SG4	45%	4%	11%	14%	20%	5%	0%	0.47%
Total	42%	9%	20%	13%	10%	4%	2%	0.26%

Table S10. Interview questions about health-related discussion networks. *Items and response options are described in English translation; originally, they were asked in Khasi, Pnar, or Garo, depending on the tribe of the village.*

No.	Question (in English)	Response options
Q15	Please name the people in your village you talk to about health-related matters. Please indicate, if you know, the family name and their nickname.	*[free response, names recorded]*
Q16	Are there any other people outside your village you talk to about health-related matters?	*[free response, names recorded]*

Table S11. Descriptive statistics of health-related discussion networks in each village. *S.d. = standard deviation.*

Village	# of nodes	# of ties	Average degree	S.d. outdegree	S.d. indegree
WK1	100	228	2.28	1.41	1.65
WK2	45	77	1.71	0.79	1.36
WK3	71	245	3.45	1.71	2.56
WJ1	315	463	1.47	1.15	1.47
WJ2	289	481	1.66	1.25	1.55
WJ3	114	160	1.40	0.93	1.12
SG1	43	86	2.00	1.35	1.54
SG2	115	194	1.69	1.07	1.26
SG3	211	238	1.13	0.81	1.13
SG4	211	361	1.71	1.05	1.19
Avg.	151	253	1.85	1.15	1.48

Table S12. The size of participants' discussion networks among non-respondents and outside their own village in each village and in total. *Avg. = Average, S.d. = Standard deviation.*

Village	Avg. no. ties to non-respondents	S.d. no. ties to non-respondents	Avg. no. ties out of village	S.d. no. ties out of village
WK1	0.69	0.87	1.30	0.78
WK2	0.60	0.66	1.22	0.50
WK3	0.55	0.49	2.01	0.48
WJ1	0.56	0.94	0.34	0.76
WJ2	0.56	0.79	0.56	0.69
WJ3	0.91	0.79	0.36	1.03
SG1	0.67	0.88	0.65	0.77
SG2	0.32	0.83	0.17	1.55
SG3	0.25	0.78	0.18	1.20
SG4	0.71	0.67	0.35	1.58
Total	0.56	0.56	0.53	0.53

Table S13. Proportion of individuals and households talking to the ASHA or the Traditional Healer about health-related matters in each village and in total. *Note: villages WJ3, SG1, SG3, and SG4 did not have their own Healer at the time of the data collection. Cell color indicates proportion of respondents/households: larger numbers marked by darker green.*

Village	% participants talk to ASHA	% participants talk to Healer	% households talk to ASHA	% households talk to Healer
WK1	35%	4%	48%	8%
WK2	27%	24%	43%	29%
WK3	65%	7%	67%	17%
WJ1	57%	4%	73%	5%
WJ2	39%	13%	51%	18%
WJ3	43%	–	55%	–
SG1	53%	–	77%	–
SG2	70%	24%	90%	42%
SG3	20%	–	35%	–
SG4	53%	–	71%	–
Total	46%	7%	50%	11%

Table S14. Average level of network exposure of individuals to the eight prevention behaviors in each village and in total. *Cell color indicates average proportion of contacts adopting a behavior: larger numbers marked by darker green.*

Village	LLINS	Covering clothes	Boots	Gloves	Insecticide cream	Coils	Vaporisers	Burning materials
WK1	79%	74%	20%	29%	8%	31%	2%	38%
WK2	76%	53%	12%	26%	3%	36%	19%	26%
WK3	90%	79%	24%	11%	21%	49%	10%	29%
WJ1	93%	80%	61%	1%	39%	63%	44%	16%
WJ2	91%	73%	34%	4%	31%	77%	2%	35%
WJ3	92%	60%	12%	0%	17%	52%	0%	14%
SG1	82%	65%	9%	3%	33%	81%	0%	11%
SG2	98%	60%	3%	0%	3%	45%	0%	35%
SG3	84%	28%	8%	2%	20%	78%	12%	3%
SG4	92%	52%	2%	1%	25%	86%	1%	26%
Total	90%	63%	25%	5%	24%	66%	13%	23%

Table S15. Median level of network exposure of individuals to the eight prevention behaviors in each village and in total. *Cell color indicates median proportion of contacts adopting a behavior: larger numbers marked by darker green.*

Village	LLINs	Covering clothes	Boots	Gloves	Insecticide cream	Coils	Vaporisers	Burning materials
WK1	100%	100%	0%	25%	0%	25%	0%	40%
WK2	100%	50%	0%	0%	0%	33%	0%	0%
WK3	100%	100%	25%	0%	20%	50%	0%	25%
WJ1	100%	100%	67%	0%	33%	67%	50%	0%
WJ2	100%	100%	33%	0%	25%	100%	0%	33%
WJ3	100%	67%	0%	0%	0%	50%	0%	0%
SG1	100%	67%	0%	0%	25%	100%	0%	0%
SG2	100%	67%	0%	0%	0%	50%	0%	33%
SG3	100%	0%	0%	0%	0%	100%	0%	0%
SG4	100%	50%	0%	0%	20%	100%	0%	25%

Table S16. **Proportion of individuals exposed to the eight prevention behaviors in their household in each village and in total.** *Cell color indicates proportion of respondents: larger numbers marked by darker green.*

Village	LLINs	Covering clothes	Boots	Gloves	Insecticide cream	Coils	Vaporisers	Burning materials
WK1	73%	61%	26%	22%	15%	44%	6%	33%
WK2	73%	56%	16%	27%	2%	42%	9%	40%
WK3	83%	72%	49%	24%	32%	63%	14%	55%
WJ1	75%	65%	50%	3%	11%	46%	20%	22%
WJ2	82%	64%	28%	6%	18%	67%	5%	28%
WJ3	52%	46%	13%	0%	15%	39%	0%	13%
SG1	77%	51%	9%	2%	12%	63%	0%	12%
SG2	90%	59%	10%	0%	7%	48%	0%	61%
SG3	86%	46%	14%	1%	35%	82%	20%	4%
SG4	88%	56%	7%	1%	8%	83%	1%	16%
Total	79%	59%	25%	6%	16%	61%	9%	25%

Table S17. Description of SAOM effects used for explaining the adoption of prevention behaviors.

	A. Effects of structure of behavior adoption	*RSiena "short names"*
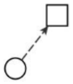	(1) Outdegree (intercept)	density
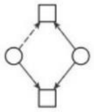	(2) Trends in village to adopt similar behaviors (4-cycles)	cycle4
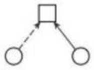	(3) Prevalence of behavior	inPop
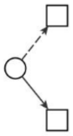	(4) Behavior carry-over (Activity of villager)	outAct
	B. Effects of individual factors	***RSiena "short names"***
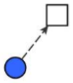	(5) Female	egoX (interaction: Female)
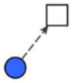	(6) Head of household	egoX (interaction: Head of Household)
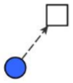	(7) Carer for a sick person	egoX (interaction: Carer for a sick person)
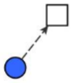	(8) Works in fields	egoX (interaction: Works in fields)
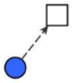	(9) Age	egoX (interaction: Age)
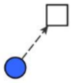	(10) Education	egoX (interaction: Education)

(Table S17 continued)

	C. Effects of health experts	***RSiena "short names"***
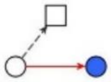	(11) Talking to the ASHA	egoX<br>(interaction: talking to the ASHA)
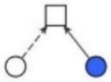	(12) ASHA's adoption of a specific behavior	altX<br>(interaction: ASHA adopts behavior)
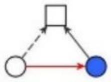	(13) ASHA's adoption of a behavior if talking to the ASHA	egoX * altX<br>(interaction: talking to the ASHA, ASHA adopts behavior)
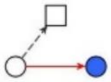	(14) Talking to the Healer	egoX<br>(interaction: talking to the Healer)
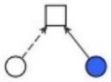	(15) Healer's adoption of a specific behavior	altX<br>(interaction: Healer adopts behavior)
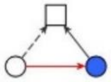	(16) Healer's adoption of a behavior if talking to the Healer	egoX * altX<br>(interaction: talking to the Healer, Healer adopts behavior)
	D. Effects of discussion network size	***RSiena "short names"***
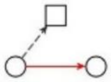	(17) Number of other villagers one talks to	outActIntn<br>(interaction: talk)
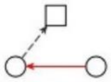	(18) Number of other villagers who talk to one	inActIntn<br>(interaction: talk)
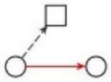	(19) Number of non-respondents one talks to	egoX<br>(interaction: outdegree to non-respodnents)
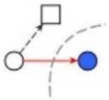	(20) Number of non-villagers one talks to	egoX<br>(interaction: outdegree outside of village)
	E. Effect of household exposure	***RSiena "short names"***
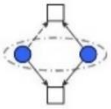	(21) Adopting the same behaviors as household members	sameXCycle4<br>(interaction: same household)
	F. Effect of network exposure	***RSiena "short names"***
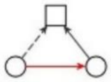	(22) Adopting the same behaviors as those one talks to	to<br>(interaction: talk)

Table S18. Description of SAOM effects used for explaining the health-related discussion network.

	A. Effects of the structure of discussion ties	*RSiena "short names"*
A1. Endogenous network effects: discussion ties with other respondents		
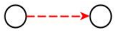	(1) Outdegree (intercept)	density
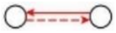	(2) Reciprocity	recip
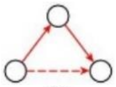	(3) Transitivity	transTrip
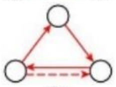	(4) Transitivity * Reciprocity	transRecTrip
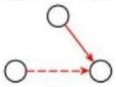	(5) Popularity of villagers	inPop
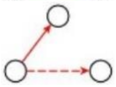	(6) Activity of villagers	outAct
A2. Exogenous network effects: discussion ties to non-respondents and people outside village		
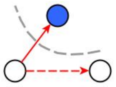	(7) Sender's Number of non-villagers they talk to	egoX (interaction: outdegree out of village)
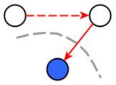	(8) Receiver's Number of non-villagers they talk to	altX (interaction: outdegree out of village)
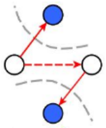	(9) Similarity in Number of non-villagers they talk to	simX (interaction: outdegree out of village)
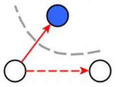	(10) Sender's Number of non-respondents they talk to	egoX (interaction: outdegree to non-respondents)
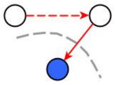	(11) Receiver's Number of non-respondents they talk to	altX (interaction: outdegree to non-respondents)
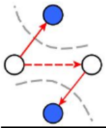	(12) Similarity in Number of non-respondents they talk to	simX (interaction: outdegree to non-respondents)
	B. Effects of individual factors	***RSiena "short names"***
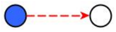	(13) Sender Female	egoX (interaction: Female)
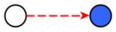	(14) Receiver Female	altX (interaction: Female)
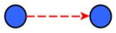	(15) Both Female	sameX (interaction: Female)
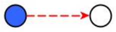	(16) Sender Head of household	egoX (interaction: Head of Household)
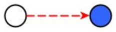	(17) Receiver Head of household	altX (interaction: Head of Household)
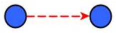	(18) Both Head of household	sameX (interaction: Head of Household)
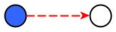	(19) Sender Carer for a sick person	egoX (interaction: Carer for a sick person)

(Table S18 continued)

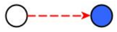	(20) Receiver Carer for a sick person	altX<br>(interaction: Carer for a sick person)
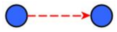	(21) Both Carers for a sick person	sameX<br>(interaction: Carer for a sick person)
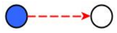	(22) Sender Works in fields	egoX<br>(interaction: Works in fields)
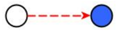	(23) Receiver Works in fields	atX<br>(interaction: Works in fields)
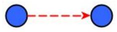	(24) Both Work in fields	sameX<br>(interaction: Works in fields)
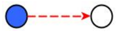	(25) Sender Age	egoX<br>(interaction: Age)
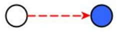	(26) Receiver Age	altX<br>(interaction: Age)
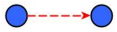	(27) Both of similar Age	simX<br>(interaction: Age)
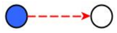	(28) Sender Education	egoX<br>(interaction: Education)
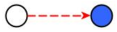	(29) Receiver Education	altX<br>(interaction: Education)
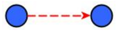	(30) Both of similar Education	simX<br>(interaction: Education)
C. Effects of ties to health experts		***RSiena "short names"***
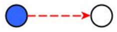	(31) Sender Talks to the ASHA	egoX<br>(interaction: talks to ASHA)
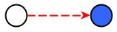	(32) Receiver Talks to the ASHA	altX<br>(interaction talks to ASHA)
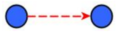	(33) Both Talk to the ASHA	sameX<br>(interaction: talks to ASHA)
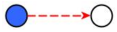	(34) Sender Talks to the Healer	egoX<br>(interaction: talks to Healer)
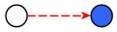	(35) Receiver Talks to the Healer	altX<br>(interaction talks to Healer)
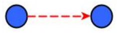	(36) Both Talk to the Healer	sameX<br>(interaction: talks to Healer)
D. Effects of number of behaviors adopted		***Rsiena "short names"***
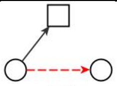	(37) Sender's Number of behaviors adopted	outActIntn<br>(interaction: behavior adoption)
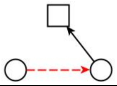	(38) Receiver's Number of behaviors adopted	outPopIntn<br>(interaction: behavior adoption)
E. Effect of household membership		***RSiena "short names"***
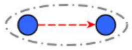	(39) Both in same Household	sameX<br>(interaction: Household)
F. Effect of adopting specific behaviors		***RSiena "short names"***
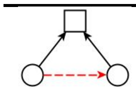	(40) Both adopting the same behavior	from<br>(interaction: behavior adoption)

Table S19. Results of score-type tests for fixed (constrained) parameters explaining the adoption of prevention behaviors in the village-level SAOMs. *Df: degrees of freedom.*

Village	Effect description	Effect number in Table S17	Parameter value	Chi-square statistic	df	p-value
WJ2	Number of other villagers who talk to one	18	0.10	0.21	1	0.648
SG1	Carer for a sick person	7	1.00	2.21	1	0.137
SG1	Talking to the ASHA	11	0.50	3.18	1	0.074
SG2	Healer's adoption of a specific behavior	15	1.00	3.06	1	0.080
SG2	Healer's adoption of a behavior if talking to the Healer	16	0.00	0.26	1	0.613
SG2	Number of other villagers one talks to	17	0.00	2.18	1	0.140

Table S20. Results of score-type tests for fixed (constrained) parameters explaining health-related discussion ties in the village-level SAOMs. *Df: degrees of freedom.*

Village	Effect description	Effect number in Table S18	Parameter value	Chi-square statistic	df	p-value
WK1	Both Talk to the Healer	30	0.00	0.05	1	0.815
WJ1	Both Talk to the Healer	30	-0.10	0.08	1	0.774
SG1	Sender Carer for a sick person	13	0.00	0.73	1	0.394
SG1	Receiver Carer for a sick person	14	0.00	1.83	1	0.177
SG1	Both Carers for a sick person	15	0.00	0.19	1	0.664
SG1	Sender Works in fields	16	0.00	0.53	1	0.467
SG1	Receiver Works in fields	17	0.00	1.37	1	0.242
SG1	Both Work in fields	18	0.00	0.05	1	0.829
SG1	Receiver's Number of behaviors adopted	36	0.00	1.83	1	0.177
SG1	Sender's Number of behaviors adopted	35	0.00	0.92	1	0.338
SG2	Both Carers for a sick person	15	0.00	0.07	1	0.799
SG2	Sender's Number of behaviors adopted	35	2.00	0.28	1	0.600
SG4	Sender Carer for a sick person	13	0.00	1.49	1	0.222
SG4	Receiver Carer for a sick person	14	0.00	0.60	1	0.440
SG4	Both Carers for a sick person	15	0.00	0.25	1	0.614

Table S21. Results of village-level SAOMs I. – model effects explaining the adoption of prevention behaviors. Parameters are log odds ratios; standard errors reported in parentheses; note "(Fixed)" means the effect was constrained to the given value during the estimation.

Dependent network: adopt (1/2)		WK1		WK2		WK3		WJ1		WJ2		WJ3		SG1		SG2		SG3		SG4	
(0)	rate parameter	3		3		3		3		3		3		3		3		3		3	
		(Fixed)		*(Fixed)*		*(Fixed)*		*(Fixed)*		*(Fixed)*		*(Fixed)*		*(Fixed)*		*(Fixed)*		*(Fixed)*		*(Fixed)*	
(1)	density	**-1.98**	**	0.03		-0.53		**-2.26**	***	**-3.04**	***	-1.11		**-2.01**		**-5.4**	***	-0.18		**-5.64**	**
		**(0.63)**		(1.92)		(1.36)		**(0.55)**		**(0.58)**		(1.37)		**(1.9)**		**(1.17)**		(1.2)		**(1.76)**	
(2)	cycle4	0.00		0.02		0.00		0.00		**-0.01**	**	0.01		-0.05		0.00		**0.01**	*	0.00	
		(0.01)		(0.03)		(0.01)		(0.00)		**(0.00)**		(0.01)		(0.03)		(0.01)		**(0.00)**		(0.00)	
(3)	inPop	0.01		0.01		0.01		**0.01**	***	**0.02**	***	0.00		**0.12**	***	**0.04**	***	0.00		**0.02**	***
		(0.01)		(0.03)		(0.02)		**(0.00)**		**(0.00)**		(0.01)		**(0.03)**		**(0.01)**		(0.01)		**(0.00)**	
(4)	outAct	0.01		-0.20		-0.10		0.03		0.06		-0.15		-0.23		-0.06		**-0.41**	***	-0.06	
		(0.07)		(0.18)		(0.11)		(0.05)		(0.06)		(0.19)		(0.31)		(0.17)		**(0.12)**		(0.15)	
(5)	egoX (female)	-0.03		-0.38		-0.31		-0.38		-0.20		-0.37		-0.57		0.10		0.05		-0.11	
		(0.26)		(0.77)		(0.36)		(0.22)		(0.17)		(0.43)		(0.59)		(0.29)		(0.24)		(0.28)	
(6)	egoX (head of household)	0.17		-1.08		0.10		-0.04		-0.19		-0.01		-0.69		-0.38		0.19		-0.62	
		(0.29)		(1.03)		(0.40)		(0.19)		(0.18)		(0.31)		(0.66)		(0.31)		(0.24)		(0.39)	
(7)	egoX (in care of sick)	-0.07		0.54		-0.06		-0.14		0.05		-0.12		1.00		1.34		-0.47		2.35	
		(0.26)		(0.9)		(0.34)		(0.2)		(0.2)		(0.37)		*(Fixed)*		(0.75)		(0.58)		(1.38)	
(8)	egoX (works in fields)	-0.03		-0.96		-0.09		0.27		0.07		-0.08		-0.33		0.77		0.04		-0.27	
		(0.28)		(0.75)		(0.29)		(0.15)		(0.13)		(0.23)		(0.81)		(0.55)		(0.21)		(0.34)	
(9)	egoX (age)	0.00		-0.02		0.00		0.00		0.00		0.00		-0.02		-0.01		0.00		0.00	
		(0.01)		(0.03)		(0.01)		(0.01)		(0.01)		(0.01)		(0.03)		(0.02)		(0.01)		(0.01)	
(10)	egoX (educ)	0.05		-0.52		0.03		0.05		0.06		-0.09		0.26		**0.41**	*	**0.19**	**	-0.13	
		(0.09)		(0.30)		(0.11)		(0.05)		(0.05)		(0.09)		(0.29)		**(0.16)**		**(0.07)**		(0.08)	
(11)	egoX (talks to Asha)	0.16		1.27		0.10		0.07		-0.38		0.27		0.50		**1.09**	*	0.46		1.42	
		(0.27)		(0.99)		(0.27)		(0.25)		(0.47)		(0.31)		*(Fixed)*		**(0.51)**		(0.26)		(0.83)	
(12)	altX (Asha adopts behavior)	0.07		0.18		-0.05		-0.01		**0.83**	**	0.01		0.32		0.65		0.19		1.17	
		(0.28)		(0.52)		(0.54)		(0.24)		**(0.29)**		(0.44)		(0.68)		(0.49)		(0.62)		(0.76)	
(13)	egoX*altX (talks to Asha *	-0.06		-0.35		0.40		0.07		0.40		1.47		0.76		-0.45		0.18		-0.52	
	Asha adopts behavior)	(0.35)		(0.92)		(0.50)		(0.28)		(0.48)		(1.07)		(0.75)		(0.56)		(0.51)		(0.86)	

* p<0.05 ** p<0.01 *** p<0.001

(Table S21 continued)

Dependent network: adopt (2/2)	WK1	WK2	WK3	WJ1	WJ2	WJ3	SG1	SG2	SG3	SG4
(14) egoX (talks to Healer)	0.17	-0.25	-0.27	0.10	0.35	0.00	0.00	0.42	0.00	0.00
	(0.53)	(0.59)	(0.54)	(0.47)	(0.24)	*(Fixed)*	*(Fixed)*	(0.32)	*(Fixed)*	*(Fixed)*
(15) altX (Healer adopts	0.13	-0.49	0.24	-0.12	-0.19	0.00	0.00	1.00	0.00	0.00
behavior)	(0.22)	(1.13)	(0.5)	(0.14)	(0.15)	*(Fixed)*	*(Fixed)*	*(Fixed)*	*(Fixed)*	*(Fixed)*
(16) egoX*altX (talks to Healer &	-0.78	1.68	-0.01	-0.07	-0.09	0.00	0.00	0.00	0.00	0.00
Healer adopts behavior)	(0.81)	(1.62)	(0.76)	(0.74)	(0.35)	*(Fixed)*	*(Fixed)*	*(Fixed)*	*(Fixed)*	*(Fixed)*
(17) outActIntn (talk)	**-0.34** *	-1.11	-0.29	**-0.72** ***	**-0.27** *	-1.24	0.13	0.00	-0.18	**-0.76** *
	**(0.15)**	(1.13)	(0.16)	**(0.2)**	**(0.12)**	(0.65)	(0.54)	*(Fixed)*	(0.32)	**(0.33)**
(18) inActIntn (talk)	-0.04	0.13	0.04	0.12	0.10	0.29	0.55	0.34	0.12	0.17
	(0.08)	(0.34)	(0.10)	(0.10)	*(Fixed)*	(0.31)	(0.58)	(0.24)	(0.18)	(0.25)
(19) egoX (outdegree to non-	0.06	0.10	0.16	0.08	0.13	0.07	0.77	0.45	0.23	-0.08
respondents)	(0.11)	-0.40	(0.19)	(0.08)	(0.07)	(0.15)	(0.58)	(0.26)	(0.24)	(0.15)
(20) egoX (outdegree out of	0.10	0.11	0.00	0.02	0.10	0.22	0.03	-0.02	0.02	0.30
village)	(0.07)	(0.21)	(0.08)	(0.10)	(0.07)	(0.16)	(0.49)	(0.25)	(0.17)	(0.19)
(21) sameXCycle4 (household)	0.08	0.01	0.06	-0.04	0.06	-0.03	-0.39	-0.64	**0.15** *	-0.22
	(0.09)	(0.3)	(0.07)	(0.06)	(0.07)	(0.14)	(0.42)	(0.46)	**(0.06)**	(0.23)
(22) to (talk)	**0.56** **	**2.81** *	**0.48** *	**1.64** ***	0.43	**1.48** **	0.46	1.44	0.59	**2.43** ***
	**(0.21)**	**(1.32)**	**(0.21)**	**(0.34)**	(0.23)	**(0.49)**	(0.56)	(0.97)	(0.31)	**(0.70)**

* p<0.05 ** p<0.01 *** p<0.001

Table S22. Results of village-level SAOMs II. – model effects explaining discussion ties. Parameters are log odds ratios; standard errors reported in parentheses; note "(Fixed)" means the effect was constrained to the given value during the estimation.

Dependent network: talk (1/3)		WK1		WK2		WK3		WJ1		WJ2		WJ3		SG1		SG2		SG3		SG4	
(0)	rate parameter	3		3		3		3		3		3		3		3		3		3	
		(Fixed)		*(Fixed)*		*(Fixed)*		*(Fixed)*		*(Fixed)*		*(Fixed)*		*(Fixed)*		*(Fixed)*		*(Fixed)*		*(Fixed)*	
(1)	density	**-6.25**	***	**-7.52**	*	**-6.01**	***	**-10.93**	***	**-9.38**	***	**-6.18**	**	**-7.23**	*	-3.54		**-6.72**	*	**-7.82**	***
		**(1.49)**		**(3.13)**		**(1.81)**		**(1.53)**		**(1.32)**		**(2.26)**		**(3.42)**		-2.10		**(3.18)**		**(2.23)**	
(2)	recip	**3.29**	***	**1.77**	*	**2.47**	***	**2.97**	***	**3.25**	***	**3.43**	***	**2.68**	**	**1.70**	**	**3.61**	***	**3.07**	***
		**(0.44)**		**(0.76)**		**(0.41)**		**(0.33)**		**(0.33)**		**(0.46)**		**(0.94)**		**-0.60**		**(0.65)**		**(0.53)**	
(3)	transTrip	**1.66**	***	**1.23**	**	**1.01**	***	**2.01**	***	**2.05**	***	**2.34**	***	**2.11**	**	**1.40**	***	**3.35**	***	**3.20**	***
		**(0.24)**		**(0.48)**		**(0.14)**		**(0.22)**		**(0.21)**		**(0.37)**		**-0.70**		**(0.34)**		**(0.74)**		**(0.35)**	
(4)	transRecTrip	**-1.48**	***	**-2.05**	*	**-0.75**	***	**-1.96**	***	**-1.91**	***	**-1.73**	**	-1.16		**-1.47**	**	**-3.28**	***	**-2.51**	***
		**(0.33)**		**(0.97)**		**(0.19)**		**(0.29)**		**(0.28)**		**(0.54)**		(0.67)		**(0.47)**		**(0.96)**		**(0.44)**	
(5)	inPop	0.05		0.14		0.03		-0.11		-0.02		-0.23		-0.68		-0.03		-0.43		**-0.36**	**
		(0.08)		(0.14)		(0.07)		(0.07)		(0.06)		(0.15)		(0.46)		(0.19)		(0.27)		**(0.13)**	
(6)	outAct	**-0.19**	*	**-1.04**	*	**-0.27**	***	**-0.30**	***	**-0.25**	**	**-0.66**	**	-0.52		**-1.02**	***	**-1.7**	***	**-0.86**	***
		**(0.09)**		**(0.45)**		**(0.08)**		**(0.09)**		**(0.08)**		**(0.23)**		(0.28)		**(0.25)**		**(0.46)**		**(0.15)**	
(7)	egoX (outdegree outside village)	**0.28**	*	0.09		0.04		0.33		**0.43**	***	-0.06		1.14		0.53		0.91		0.26	
		**(0.13)**		(0.30)		(0.10)		(0.20)		**(0.13)**		(0.32)		(0.59)		(0.61)		(0.57)		(0.25)	
(8)	altX (outdegree outside village)	-0.08		-0.04		0.04		-0.04		0.05		0.09		-0.67		-0.01		0.37		0.21	
		(0.09)		(0.20)		(0.09)		(0.15)		(0.10)		(0.24)		(0.53)		(0.46)		(0.43)		(0.18)	
(9)	simX (outdegree outside village)	0.29		0.35		0.19		-0.17		1.02		0.07		0.89		-1.13		**3.43**	**	-0.03	
		(0.94)		(1.09)		(0.68)		(0.43)		(0.62)		(0.70)		(0.78)		(1.38)		**(1.33)**		(0.75)	
(10)	egoX (outdegree to non-respondents)	0.17		0.70		-0.19		0.06		-0.08		-0.29		0.79		**-1.26**	*	-1.63	*	-0.06	
		(0.21)		(0.49)		(0.27)		(0.15)		(0.17)		(0.27)		(0.52)		**(0.59)**		(0.77)		(0.2)	
(11)	altX (outdegree to non-respondents)	0.17		0.09		-0.16		0.08		-0.04		0.04		-0.75		-0.32		-0.19		**0.34**	**
		(0.17)		(0.28)		(0.23)		(0.10)		(0.13)		(0.19)		(0.63)		(0.38)		(0.53)		**(0.13)**	
(12)	simX (outdegree to non-respondents)	0.27		-0.30		-0.12		0.23		0.82		-0.03		-0.19		0.70		1.70		0.50	
		(0.50)		(0.89)		(0.38)		(0.42)		(0.49)		(0.65)		(1.58)		(1.41)		(1.34)		(0.54)	

* p<0.05 ** p<0.01 *** p<0.001

(Table S22 continued)

Dependent network: talk (2/3)		WK1	WK2	WK3	WJ1	WJ2	WJ3	SG1	SG2	SG3	SG4
(13)	egoX (female)	-0.37	0.75	0.09	0.02	0.53	-0.72	1.22	0.33	-0.35	-0.21
		(0.48)	(0.97)	(0.47)	(0.41)	(0.35)	(0.58)	(0.87)	(0.53)	(0.53)	(0.33)
(14)	altX (female)	0.38	0.12	0.80	**1.06** ***	**0.74** **	**1.04** *	0.58	0.54	**1.20** **	0.49
		(0.36)	(0.59)	(0.43)	**(0.27)**	**(0.26)**	**(0.48)**	-0.70	(0.35)	**-0.40**	(0.27)
(15)	sameX (female)	0.17	0.61	0.06	0.18	0.25	**0.85** *	-0.19	0.32	0.31	0.16
		(0.29)	(0.42)	(0.26)	(0.21)	(0.21)	**(0.36)**	-0.50	(0.28)	(0.32)	(0.24)
(16)	egoX (head of household)	-0.42	0.10	-0.30	-0.41	-0.27	0.35	-0.14	-0.96	-0.82	**1.08** *
		(0.49)	(1.09)	(0.51)	(0.37)	(0.36)	(0.53)	(0.87)	(0.65)	(0.66)	**(0.48)**
(17)	altX (head of household)	0.33	-0.44	0.44	0.04	0.05	-0.26	1.43	0.56	**1.00** *	**1.75** ***
		(0.39)	(0.73)	(0.44)	(0.22)	(0.23)	(0.38)	(0.96)	(0.39)	**(0.45)**	**(0.44)**
(18)	sameX (head of household)	0.05	-0.65	0.48	-0.03	0.02	-0.02	0.13	-0.04	-0.38	**-0.71** *
		(0.27)	(0.49)	(0.29)	(0.18)	(0.17)	(0.26)	(0.73)	(0.29)	(0.34)	**(0.36)**
(19)	egoX (in care of sick)	0.45	1.76	0.42	0.70	0.52	0.64	0.00	0.85	2.08	0.00
		(0.52)	(0.98)	(0.46)	(0.56)	(0.54)	(0.84)	*(Fixed)*	(1.05)	-1.70	*(Fixed)*
(20)	altX (in care of sick)	0.47	0.93	0.28	0.75	0.79	-0.18	0.00	-0.99	0.51	0.00
		(0.50)	(0.66)	(0.37)	(0.54)	(0.51)	(0.76)	*(Fixed)*	(0.77)	-1.60	*(Fixed)*
(21)	sameX (in care of sick)	0.16	0.35	-0.14	-0.32	-0.42	-0.32	0.00	0.00	-0.86	0.00
		(0.39)	(0.61)	(0.28)	(0.48)	(0.49)	(0.65)	*(Fixed)*	*(Fixed)*	(0.97)	*(Fixed)*
(22)	egoX (works in fields)	-0.61	0.97	0.02	-0.05	0.24	-0.18	0.00	**-1.72**	-1.03	0.03
		(0.52)	(0.99)	(0.34)	(0.33)	(0.27)	(0.39)	*(Fixed)*	**(1.02)**	(0.57)	(0.49)
(23)	altX (works in fields)	-0.37	-0.11	0.46	-0.05	0.26	0.13	0.00	-0.04	0.26	0.13
		(0.51)	(0.66)	(0.32)	(0.24)	(0.19)	(0.28)	*(Fixed)*	(0.78)	(0.41)	(0.39)
(24)	sameX (works in fields)	0.04	-0.47	-0.08	0.12	0.07	0.19	0.00	0.16	0.08	-0.47
		(0.37)	(0.53)	(0.23)	(0.21)	(0.17)	(0.23)	*(Fixed)*	(0.66)	(0.31)	(0.34)
(25)	egoX (age)	0.00	-0.02	0.02	-0.01	0.01	0.01	0.00	0.04	0.00	-0.01
		(0.01)	(0.03)	(0.02)	(0.01)	(0.01)	(0.02)	(0.04)	(0.02)	(0.03)	(0.01)
(26)	altX (age)	0.01	0.04	0.03	**0.05** ***	**0.04** ***	**0.04** *	0.04	**0.04** **	**0.09** **	**0.03** *
		(0.01)	(0.02)	(0.01)	**(0.01)**	**(0.01)**	**(0.02)**	(0.04)	**(0.01)**	**(0.03)**	**(0.01)**

* p<0.05 ** p<0.01 *** p<0.001

(Table S22 continued)

Dependent network: talk (3/3)	WK1	WK2	WK3	WJ1	WJ2	WJ3	SG1	SG2	SG3	SG4
(27) simX (age)	0.28	1.18	0.79	0.31	**0.86** *	**1.26** *	-1.92	**1.96** **	1.06	**1.49** *
	(0.58)	(0.92)	(0.69)	(0.44)	**(0.44)**	**(0.63)**	(1.86)	**(0.76)**	(0.87)	**(0.71)**
(28) egoX (education)	0.02	0.18	0.13	-0.08	0.02	-0.11	0.04	**-0.53** *	-0.02	-0.05
	(0.15)	(0.30)	(0.14)	(0.10)	(0.10)	(0.19)	(0.27)	**(0.22)**	(0.20)	(0.10)
(29) altX (education)	0.00	-0.04	0.04	0.04	-0.01	-0.04	-0.6	0.18	-0.02	0.08
	(0.13)	(0.19)	(0.11)	(0.08)	(0.07)	(0.14)	(0.38)	(0.19)	(0.13)	(0.08)
(30) simX (education)	0.61	-0.05	-0.35	0.79	-0.35	-0.25	0.20	0.40	-0.06	0.79
	(0.71)	(0.91)	(0.49)	(0.44)	(0.39)	(0.8)	(0.82)	(0.47)	(0.63)	(0.42)
(31) egoX (talks to Asha)	0.19	1.11	-0.48	-0.44	-0.21	0.21	-0.50	-0.34	-0.71	**2.29** ***
	(0.32)	(1.00)	(0.37)	(0.24)	(0.25)	(0.42)	(0.96)	(0.57)	(0.75)	**(0.59)**
(32) altX (talks to Asha)	-0.07	-0.50	0.09	**0.39** *	0.30	-0.34	0.16	-0.17	0.33	-0.56
	(0.26)	(0.58)	(0.29)	**(0.18)**	(0.17)	(0.29)	(0.81)	(0.44)	(0.50)	(0.40)
(33) sameX (talks to Asha)	0.06	-0.29	-0.01	0.20	0.04	-0.01	-0.44	0.31	-0.01	0.08
	(0.24)	(0.54)	(0.26)	(0.15)	(0.15)	(0.24)	(0.58)	(0.35)	(0.40)	(0.27)
(34) egoX (talks to Healer)	-0.43	0.93	-0.25	**1.02** *	0.09	0.00	0.00	0.57	0.00	0.00
	(0.93)	(0.96)	(0.82)	**(0.51)**	(0.35)	*(Fixed)*	*(Fixed)*	(0.62)	*(Fixed)*	*(Fixed)*
(35) altX (talks to Healer)	0.25	0.10	0.15	-0.70	0.42	0.00	0.00	0.31	0.00	0.00
	(0.63)	(0.60)	(0.78)	(0.47)	(0.30)	*(Fixed)*	*(Fixed)*	(0.40)	*(Fixed)*	*(Fixed)*
(36) sameX (talks to Healer)	0.00	0.36	-0.18	-0.10	**0.64** *	0.00	0.00	0.64	0.00	0.00
	(Fixed)	(0.59)	(0.78)	*(Fixed)*	**(0.27)**	*(Fixed)*	*(Fixed)*	(0.34)	*(Fixed)*	*(Fixed)*
(37) outActIntn (behavior adoption)	**-0.68** *	-0.80	-0.08	**-0.67** *	-0.07	-1.05	0.00	2.00	0.83	-0.63
	**(0.31)**	(0.73)	(0.33)	**(0.29)**	(0.29)	(0.72)	*(Fixed)*	*(Fixed)*	(1.14)	(0.66)
(38) outPopIntn (behavior adoption)	-0.41	-0.89	-0.13	**-0.54** *	-0.03	-0.20	0.00	0.17	0.72	-0.11
	(0.28)	(0.66)	(0.27)	**(0.22)**	(0.25)	(0.41)	*(Fixed)*	(0.60)	(0.74)	(0.53)
(39) sameX (household)	**2.89** ***	**2.64** **	**1.55** ***	**3.09** ***	**3.00** ***	**2.47** ***	**4.83** *	**4.93** ***	**4.27** ***	**3.55** ***
	**(0.58)**	**(0.83)**	**(0.40)**	**(0.37)**	**(0.33)**	**(0.52)**	**(2.03)**	**(0.77)**	**(0.62)**	**(0.48)**
(40) from (behavior adoption)	0.97	1.75	0.55	**1.64** ***	0.22	0.90	2.20	0.24	-0.18	0.91
	(0.53)	(1.08)	(0.37)	**(0.39)**	(0.43)	(0.77)	(1.68)	(0.77)	(0.70)	(0.81)

* p<0.05 ** p<0.01 *** p<0.001

Table S23. Full results of meta-analyses of village-level SAOMs I. – model effects explaining the adoption of prevention behaviors. μ: estimated mean of the village-level parameters; se: standard error of μ; p: p-value of the test μ=0; τ: estimated between-village standard deviation of parameters; Q: Cochran's Q (variability of village-level parameters around the fixed-effects mean); Qp: p-value of the test Q=0; n: number of village-level models use in the meta-analysis.

Dependent network: adopt		mu	se	p	tau	Q	Qp	n
(0)	rate parameter	3.00			*(Fixed)*			
(1)	density	**-2.28**	**0.52**	**0.000**	1.17	19.55	0.021	10
(2)	cycle4	0.00	0.00	0.884	0.00	20.25	0.016	10
(3)	inPop	**0.01**	**0.00**	**0.000**	0.00	20.93	0.013	10
(4)	outAct	-0.07	0.05	0.172	0.11	16.65	0.054	10
(5)	egoX (female)	-0.16	0.09	0.061	0.00	3.93	0.916	10
(6)	egoX (head of household)	-0.09	0.09	0.302	0.00	7.27	0.609	10
(7)	egoX (in care of sick)	-0.02	0.11	0.849	0.00	7.88	0.445	9
(8)	egoX (works in fields)	0.06	0.07	0.407	0.00	7.25	0.611	10
(9)	egoX (age)	0.00	0.00	0.772	0.00	2.34	0.985	10
(10)	egoX (educ)	0.04	0.04	0.327	0.09	21.25	0.012	10
(11)	egoX (talks to Asha)	**0.25**	**0.11**	**0.024**	0.00	9.19	0.326	9
(12)	altX (Asha adopts behavior)	0.28	0.14	0.054	0.19	8.41	0.494	10
(13)	egoX*altX (talks to Asha * Asha adopts)	0.11	0.16	0.475	0.00	5.14	0.822	10
(14)	egoX (talks to Healer)	0.23	0.16	0.140	0.00	2.20	0.821	6
(15)	altX (Healer adopts behavior)	-0.09	0.09	0.308	0.00	2.04	0.728	5
(16)	egoX*altX (talks to Healer * Healer adopts)	-0.10	0.27	0.703	0.00	1.93	0.748	5
(17)	outActIntn (talk)	**-0.38**	**0.07**	**0.000**	0.03	8.77	0.362	9
(18)	inActIntn (talk)	0.07	0.05	0.180	0.00	4.73	0.786	9
(19)	egoX (outdegree to non-respondents)	**0.10**	**0.04**	**0.014**	0.00	5.36	0.801	10
(20)	egoX (outdegree out of village)	**0.08**	**0.04**	**0.028**	0.00	3.74	0.928	10
(21)	sameXCycle4 (household)	0.04	0.03	0.200	0.03	9.54	0.389	10
(22)	to (talk)	**0.93**	**0.21**	**0.000**	0.48	22.67	0.007	10

Table S24. Full results of meta-analyses of village-level SAOMs II. – model effects explaining discussion ties. For an explanation of column names, see the caption of Table S23.

Dependent network: talk		mu	se	p	tau	Q	Qp	n
(0)	rate parameter	3.00	*(Fixed)*					
(1)	density	**-7.41**	**0.80**	**0.000**	1.48	12.38	0.193	10
(2)	recip	**2.95**	**0.16**	**0.000**	0.15	11.77	0.227	10
(3)	transTrip	**1.95**	**0.23**	**0.000**	0.63	57.33	0.000	10
(4)	transRecTrip	**-1.68**	**0.21**	**0.000**	0.48	28.95	0.001	10
(5)	inPop	-0.07	0.05	0.139	0.09	16.52	0.057	10
(6)	outAct	**-0.56**	**0.12**	**0.000**	0.32	37.47	0.000	10
(7)	egoX (outdegree outside village)	**0.26**	**0.08**	**0.001**	0.12	11.45	0.246	10
(8)	altX (outdegree outside village)	0.01	0.05	0.851	0.00	4.95	0.839	10
(9)	simX (outdegree outside village)	0.31	0.23	0.184	0.00	10.10	0.342	10
(10)	egoX (outdegree to non-respondents)	-0.03	0.08	0.690	0.00	16.16	0.064	10
(11)	altX (outdegree to non-respondents)	0.07	0.06	0.257	0.07	9.48	0.394	10
(12)	simX (outdegree to non-respondents)	0.26	0.18	0.165	0.00	4.43	0.881	10
(13)	egoX (female)	0.03	0.15	0.831	0.00	8.13	0.521	10
(14)	altX (female)	**0.72**	**0.11**	**0.000**	0.00	6.40	0.699	10
(15)	sameX (female)	**0.24**	**0.09**	**0.005**	0.00	5.26	0.811	10
(16)	egoX (head of household)	-0.17	0.19	0.383	0.29	11.02	0.274	10
(17)	altX (head of household)	**0.41**	**0.20**	**0.038**	0.46	21.01	0.013	10
(18)	sameX (head of household)	-0.04	0.08	0.630	0.00	9.34	0.406	10
(19)	egoX (in care of sick)	**0.63**	**0.23**	**0.006**	0.00	2.48	0.929	8
(20)	altX (in care of sick)	**0.40**	**0.20**	**0.047**	0.00	5.64	0.583	8
(21)	sameX (in care of sick)	-0.14	0.17	0.429	0.00	2.38	0.881	7
(22)	egoX (works in fields)	-0.08	0.14	0.567	0.00	9.19	0.326	9
(23)	altX (works in fields)	0.15	0.11	0.161	0.00	3.36	0.909	9
(24)	sameX (works in fields)	0.02	0.09	0.783	0.00	4.03	0.854	9
(25)	egoX (age)	0.00	0.01	0.738	0.00	7.51	0.584	10
(26)	altX (age)	**0.04**	**0.00**	**0.000**	0.00	9.44	0.398	10
(27)	simX (age)	**0.83**	**0.20**	**0.000**	0.00	8.27	0.508	10
(28)	egoX (education)	-0.03	0.04	0.543	0.00	7.77	0.557	10
(29)	altX (education)	0.02	0.03	0.505	0.00	4.69	0.860	10
(30)	simX (education)	0.22	0.19	0.251	0.23	8.04	0.530	10
(31)	egoX (talks to Asha)	0.05	0.26	0.853	0.63	23.91	0.004	10
(32)	altX (talks to Asha)	0.06	0.12	0.638	0.19	10.97	0.278	10
(33)	sameX (talks to Asha)	0.07	0.08	0.356	0.00	2.65	0.977	10
(34)	egoX (talks to Healer)	0.34	0.24	0.147	0.00	3.94	0.558	6
(35)	altX (talks to Healer)	0.15	0.20	0.449	0.12	4.22	0.518	6
(36)	sameX (talks to Healer)	**0.56**	**0.19**	**0.004**	0.00	1.17	0.760	4
(37)	outActIntn (behavior adoption)	**-0.42**	**0.15**	**0.005**	0.11	6.28	0.508	8
(38)	outPopIntn (behavior adoption)	**-0.26**	**0.11**	**0.021**	0.00	6.25	0.619	9
(39)	sameX (household)	**3.11**	**0.31**	**0.000**	0.77	27.19	0.001	10
(40)	from (behavior adoption)	**0.78**	**0.23**	**0.001**	0.36	10.92	0.281	10

Table S25. Goodness of Fit p-values of the full SAOMs on different sets of network statistics in each village. *Values closer to one represent good fit, while those closer to 0 poorer fit; exact 0 values would represent inadequate fit, which is not observed in any of the cases; cell color indicates the size of the p-value: larger numbers marked by darker green.*

	WK1	WK2	WK3	WJ1	WJ2	WJ3	SG1	SG2	SG3	SG4
(1) adopt: outdegree distribution	0.7112	0.9792	0.0882	0.9270	0.7734	0.9420	0.7866	0.1124	0.0182	0.6314
(2) adopt: indegree distribution	0.2290	0.5628	0.5470	0.0046	0.5766	0.0036	0.8388	0.3348	0.2726	0.7768
(3) mixed triad census (all types)	0.9574	0.7404	0.8746	0.0002	0.7348	0.0044	0.7240	0.8882	0.6188	0.0316
(4) mixed triad census (similarity)	0.9952	0.9358	0.9872	0.7376	0.5578	0.2412	0.5848	0.8420	0.9018	0.9150
(5) talk: outdegree distribution	0.8760	0.0696	0.9468	0.5172	0.9944	0.3724	0.9212	0.1346	0.9284	0.9872
(6) talk: indegree distribution	0.8984	0.6274	0.8916	0.9890	0.4352	0.3754	0.9558	0.3724	0.7080	0.9358
(7) talk: triad census	0.4168	0.6650	0.9910	0.0804	0.2360	0.5066	0.3314	0.9950	0.6648	0.1968
(8) talk: geodesic distance distribution	0.8230	0.7302	0.9930	0.3432	0.1246	0.9144	0.5920	0.5152	0.6342	0.6664
(9) talk: clique census (up to size 5)	0.9794	0.5096	0.9850	0.5278	0.2892	0.5034	0.9918	0.1430	0.3838	0.6362

Table S26. Goodness of fit p-values for the mixed triad census (line 3 in Table S25) in seven nested SAOM specifications in each village. *Values closer to one represent good fit, while those closer to 0 poorer fit; exact 0 values would represent inadequate fit; cell color indicates the size of the p-value: larger numbers marked by darker green.*

	WK1	WK2	WK3	WJ1	WJ2	WJ3	SG1	SG2	SG3	SG4	Mean
(1) Structural model	0.0036	0	0.0002	0	0	0	0.8136	0.0558	0	0	0.0873
(2) Individual model	0.0076	0.0002	0.0004	0	0.0004	0	0.9198	0.4844	0	0	0.1413
(3) Health experts model	0.0040	0.0016	0.0002	0	0.0006	0.0004	0.7834	0.6944	0	0	0.1485
(4) Network size model	0.0050	0.0004	0.0002	0	0.0026	0.0002	0.9940	0.9734	0	0	0.1976
(5) Household exposure model	0.4758	0.1630	0.8352	0	0.4282	0.0036	0.9200	0.9212	0.4700	0.0228	0.4240
(6) Network exposure model	0.8256	0.2724	0.5386	0	0.6286	0.0028	0.9952	0.9866	0.2348	0.0158	0.4500
(7) Full model	0.9574	0.7404	0.8746	0.0002	0.7348	0.0044	0.7240	0.8882	0.6188	0.0316	0.5574

Table S27. Results of t-tests comparing the average GoF p-values of seven nested SAOM specifications. *The first row reports a two-sided, one-sample t-test of the mean of the Structural model against 0. All other rows report one-sided, paired-sample t-tests of the given models; df: degrees of freedom.*

Model A	Model B	Mean A	Mean B	Difference of means	t-stat.	df	p	
(1) Structural		0.09	0.00	0.09	1.08	9	0.308	
(2) Individual	(1) Structural	0.14	0.09	0.05	1.26	9	0.120	
(3) Health experts	(2) Individual	0.15	0.14	0.01	0.27	9	0.395	
(4) Network size	(3) Health experts	0.20	0.15	0.05	1.49	9	0.085	
(5) Household exposure	(4) Network size	0.42	0.20	0.23	2.34	9	0.022	*
(6) Network exposure	(4) Network size	0.45	0.20	0.25	2.60	9	0.014	*
(7) Full	(4) Network size	0.56	0.20	0.36	2.46	9	0.018	*
(6) Network exposure	(5) Household exposure	0.45	0.42	0.03	0.44	9	0.664	
(7) Full	(5) Household exposure	0.56	0.42	0.13	1.72	9	0.060	
(7) Full	(6) Network exposure	0.56	0.45	0.11	1.48	9	0.087	

* p<0.05 ** p<0.01 *** p<0.001

Table S28. SAOM meta-analysis results from three models with different rate parameters: 3-3 (the results reported in the main text and above), 5-5, and 8-8. μ: estimated mean of the village-level parameters; se: standard error of μ; p: p-value of the test μ=0; n: number of village-level models use in the meta-analysis; heterogeneity statistics for models with rates 5-5 and 8-8 are available from the authors.

Dependent network: adopt		mu	se	p	n	mu	se	p	n	mu	se	p	n
(0)	rate parameter	3		*(Fixed)*		5		*(Fixed)*		8		*(Fixed)*	
(1)	density	**-2.28**	**0.52**	**0.000**	10	**-2.00**	**0.40**	**0.000**	10	-1.48	0.78	0.057	4
(2)	cycle4	0.00	0.00	0.884	10	0.00	0.00	0.954	10	0.00	0.00	0.491	4
(3)	inPop	**0.01**	**0.00**	**0.000**	10	**0.01**	**0.00**	**0.000**	10	**0.01**	**0.00**	**0.000**	4
(4)	outAct	-0.07	0.05	0.172	10	-0.08	0.05	0.102	10	-0.02	0.07	0.809	4
(5)	egoX (female)	-0.16	0.09	0.061	10	**-0.19**	**0.09**	**0.025**	10	-0.25	0.13	0.054	4
(6)	egoX (head of household)	-0.09	0.09	0.302	10	-0.07	0.09	0.408	10	-0.08	0.18	0.646	4
(7)	egoX (in care of sick)	-0.02	0.11	0.849	9	-0.08	0.10	0.431	8	0.01	0.14	0.969	4
(8)	egoX (works in fields)	0.06	0.07	0.407	10	0.06	0.07	0.352	10	0.02	0.10	0.872	4
(9)	egoX (age)	0.00	0.00	0.772	10	0.00	0.00	0.949	10	0.00	0.00	0.628	4
(10)	egoX (educ)	0.04	0.04	0.327	10	0.05	0.04	0.186	10	0.06	0.04	0.147	4
(11)	egoX (talks to Asha)	**0.25**	**0.11**	**0.024**	9	**0.24**	**0.10**	**0.020**	9	0.13	0.17	0.436	4
(12)	altX (Asha adopts behavior)	0.28	0.14	0.054	10	**0.27**	**0.13**	**0.041**	10	0.36	0.19	0.057	4
(13)	egoX*altX (talks to Asha *Asha adopts)	0.11	0.16	0.475	10	0.14	0.14	0.336	10	0.02	0.20	0.941	4
(14)	egoX (talks to Healer)	0.23	0.16	0.140	6	0.21	0.15	0.158	6	0.00	0.21	0.993	4
(15)	altX (Healer adopts behavior)	-0.09	0.09	0.308	5	-0.08	0.08	0.351	5	0.01	0.13	0.959	4
(16)	egoX*altX (talks to Healer * Healer adopts)	-0.10	0.27	0.703	5	-0.12	0.23	0.617	5	-0.19	0.32	0.553	4
(17)	outActIntn (talk)	**-0.38**	**0.07**	**0.000**	9	**-0.41**	**0.09**	**0.000**	6	**-0.45**	**0.15**	**0.002**	4
(18)	inActIntn (talk)	0.07	0.05	0.180	9	0.09	0.06	0.137	9	0.10	0.11	0.349	3
(19)	egoX (outdegree to non-respondents)	**0.10**	**0.04**	**0.014**	10	**0.09**	**0.04**	**0.016**	10	**0.11**	**0.06**	**0.048**	4
(20)	egoX (outdegree out of village)	**0.08**	**0.04**	**0.028**	10	**0.08**	**0.03**	**0.013**	10	0.07	0.05	0.148	4
(21)	sameXCycle4 (household)	0.04	0.03	0.200	10	0.03	0.04	0.469	10	0.02	0.05	0.645	4
(22)	to (talk)	**0.93**	**0.21**	**0.000**	10	**0.97**	**0.23**	**0.000**	10	**0.66**	**0.15**	**0.000**	4

(Table S28 continued)

Dependent network: talk (1/2)		mu	se	p	n	mu	se	p	n	mu	se	p	n
(0)	rate parameter	3		*(Fixed)*		5		*(Fixed)*		8		*(Fixed)*	
(1)	density	**-7.41**	**0.80**	**0.000**	10	**-7.13**	**1.14**	**0.000**	10	**-7.14**	**1.57**	**0.000**	4
(2)	recip	**2.95**	**0.16**	**0.000**	10	**2.74**	**0.24**	**0.000**	10	**2.54**	**0.44**	**0.000**	4
(3)	transTrip	**1.95**	**0.23**	**0.000**	10	**1.89**	**0.23**	**0.000**	10	**1.44**	**0.25**	**0.000**	4
(4)	transRecTrip	**-1.68**	**0.21**	**0.000**	10	**-1.74**	**0.21**	**0.000**	10	**-1.44**	**0.32**	**0.000**	4
(5)	inPop	-0.07	0.05	0.139	10	-0.10	0.05	0.052	10	0.00	0.07	0.957	4
(6)	outAct	**-0.56**	**0.12**	**0.000**	10	**-0.65**	**0.15**	**0.000**	10	**-0.34**	**0.07**	**0.000**	4
(7)	egoX (outdegree outside village)	0.01	0.05	0.851	10	0.00	0.04	0.910	10	-0.01	0.05	0.840	4
(8)	altX (outdegree outside village)	**0.26**	**0.08**	**0.001**	10	**0.28**	**0.08**	**0.001**	10	0.21	0.12	0.073	4
(9)	simX (outdegree outside village)	0.31	0.23	0.184	10	**0.41**	**0.20**	**0.040**	10	**0.64**	**0.29**	**0.029**	4
(10)	egoX (outdegree to non-respondents)	0.07	0.06	0.257	10	0.04	0.07	0.573	10	0.05	0.07	0.478	4
(11)	altX (outdegree to non-respondents)	-0.03	0.08	0.690	10	-0.08	0.07	0.284	10	0.01	0.12	0.946	4
(12)	simX (outdegree to non-respondents)	0.26	0.18	0.165	10	0.28	0.17	0.102	10	0.11	0.21	0.583	4
(13)	egoX (female)	0.03	0.15	0.831	10	-0.01	0.15	0.924	10	0.18	0.28	0.530	4
(14)	altX (female)	**0.72**	**0.11**	**0.000**	10	**0.70**	**0.11**	**0.000**	10	0.41	0.26	0.116	4
(15)	sameX (female)	**0.24**	**0.09**	**0.005**	10	**0.21**	**0.08**	**0.009**	10	0.10	0.12	0.411	4
(16)	egoX (head of household)	-0.17	0.19	0.383	10	-0.20	0.21	0.331	10	-0.16	0.23	0.489	4
(17)	altX (head of household)	**0.41**	**0.20**	**0.038**	10	0.33	0.18	0.065	10	-0.02	0.17	0.897	4
(18)	sameX (head of household)	-0.04	0.08	0.630	10	-0.04	0.08	0.617	10	0.08	0.13	0.519	4
(19)	egoX (in care of sick)	**0.63**	**0.23**	**0.006**	8	**0.64**	**0.23**	**0.005**	8	0.49	0.30	0.105	4
(20)	altX (in care of sick)	**0.40**	**0.20**	**0.047**	8	**0.49**	**0.19**	**0.009**	8	**0.58**	**0.22**	**0.008**	4
(21)	sameX (in care of sick)	-0.14	0.17	0.429	7	-0.10	0.15	0.505	7	0.06	0.17	0.719	4
(22)	egoX (works in fields)	-0.08	0.14	0.567	9	-0.05	0.13	0.670	9	-0.01	0.22	0.974	4
(23)	altX (works in fields)	0.15	0.11	0.161	9	0.16	0.10	0.110	9	0.07	0.06	0.277	4
(24)	sameX (works in fields)	0.02	0.09	0.783	9	0.03	0.08	0.706	9	-0.04	0.10	0.708	4

(Table S28 continued)

Dependent network: talk (2/2)		mu	se	p	n	mu	se	p	n	mu	se	p	n
(25)	egoX (age)	0.00	0.01	0.738	10	0.00	0.01	0.809	10	0.00	0.01	0.710	4
(26)	altX (age)	**0.04**	**0.00**	**0.000**	10	**0.04**	**0.01**	**0.000**	10	**0.03**	**0.01**	**0.002**	4
(27)	simX (age)	**0.83**	**0.20**	**0.000**	10	**0.75**	**0.19**	**0.000**	10	**0.54**	**0.25**	**0.033**	4
(28)	egoX (education)	-0.03	0.04	0.543	10	-0.02	0.04	0.590	10	0.07	0.06	0.304	4
(29)	altX (education)	0.02	0.03	0.505	10	0.02	0.03	0.529	10	0.07	0.06	0.224	4
(30)	simX (education)	0.22	0.19	0.251	10	0.20	0.18	0.284	10	-0.25	0.20	0.197	4
(31)	egoX (talks to Asha)	0.05	0.26	0.853	10	-0.03	0.27	0.915	10	-0.08	0.24	0.751	4
(32)	altX (talks to Asha)	0.06	0.12	0.638	10	0.04	0.12	0.768	10	0.06	0.13	0.631	4
(33)	sameX (talks to Asha)	0.07	0.08	0.356	10	0.09	0.07	0.185	10	0.02	0.09	0.789	4
(34)	egoX (talks to Healer)	0.34	0.24	0.147	6	0.32	0.23	0.156	6	-0.02	0.33	0.956	4
(35)	altX (talks to Healer)	0.15	0.20	0.449	6	0.17	0.17	0.310	6	0.02	0.02	0.294	4
(36)	sameX (talks to Healer)	**0.56**	**0.19**	**0.004**	4	**0.53**	**0.17**	**0.002**	4	**0.47**	**0.20**	**0.020**	3
(37)	outActIntn (behavior adoption)	**-0.42**	**0.15**	**0.005**	8	-0.57	0.32	0.074	4	-0.76	0.77	0.324	2
(38)	outPopIntn (behavior adoption)	**-0.26**	**0.11**	**0.021**	9	-0.35	0.19	0.061	8	-0.60	0.55	0.276	3
(39)	sameX (household)	**3.11**	**0.31**	**0.000**	10	**3.20**	**0.41**	**0.000**	8	**2.55**	**0.63**	**0.000**	3
(40)	from (behavior adoption)	**0.78**	**0.23**	**0.001**	10	**1.13**	**0.30**	**0.000**	10	**1.24**	**0.56**	**0.027**	4

7 References

1. Kessler, A. *et al.* Malaria in Meghalaya: a systematic literature review and analysis of data from the National Vector-Borne Disease Control Programme. *Malar J* **17**, 411 (2018).
2. Smith, J. A., Moody, J. & Morgan, J. H. Network sampling coverage II: The effect of non-random missing data on network measurement. *Social Networks* **48**, 78–99 (2017).
3. Albert, S., Nongrum, M., Webb, E. L., Porter, J. D. H. & Kharkongor, G. C. Medical pluralism among indigenous peoples in northeast India - implications for health policy. *Trop Med Int Health* **20**, 952–960 (2015).
4. Pedersen, T. L. An Implementation of Grammar of Graphics for Graphs and Networks. https://ggraph.data-imaginist.com/.
5. Schoch, D. graphlayouts: Layout algorithms for networkvisualizations in R. *JOSS* **8**, 5238 (2023).
6. Ripley, R. M., Snijders, T. A. B., Boda, Z., Vörös, A. & Preciado, P. Manual for RSiena (v. 1.3.6). (2022).
7. Viechtbauer, W. Conducting Meta-Analyses in *R* with the **metafor** Package. *J. Stat. Soft.* **36**, (2010).
8. Block, P. Reciprocity, transitivity, and the mysterious three-cycle. *Social Networks* **40**, 163–173 (2015).
9. Hauck, W. W. & Donner, A. Wald's Test as Applied to Hypotheses in Logit Analysis. *Journal of the American Statistical Association* **72**, 851 (1977).
10. Lospinoso, J. & Snijders, T. A. Goodness of fit for stochastic actor-oriented models. *Methodological Innovations* **12**, 205979911988428 (2019).
11. Hollway, J., Lomi, A., Pallotti, F. & Stadtfeld, C. Multilevel social spaces: The network dynamics of organizational fields. *Net Sci* **5**, 187–212 (2017).
12. Mahalanobis, P. C. On the generalized distance in statistics. in (1936).
13. Snijders, T. A. B. & Steglich, C. E. G. Representing Micro–Macro Linkages by Actor-based Dynamic Network Models. *Sociological Methods & Research* **44**, 222–271 (2015).
14. Simpson, C. R. Social Support and Network Formation in a Small-Scale Horticulturalist Population. *Sci Data* **9**, 570 (2022).
